# Synthesis and Evaluation of Serinolamide Derivatives as Sphingosine-1-Phosphate-1 (S1P_1_) Receptor Agonists

**DOI:** 10.3390/molecules27092818

**Published:** 2022-04-28

**Authors:** Sun Jun Park, Jushin Kim, Jaehwan Kim, Yoowon Kim, Elijah Hwejin Lee, Hyeon Jeong Kim, Siwon Kim, Byungeun Kim, Rium Kim, Ji Won Choi, Jong-Hyun Park, Ki Duk Park

**Affiliations:** 1 Convergence Research Center for Brain Science, Korea Institute of Science & Technology (KIST), Seoul 02792, Korea; h18502@kist.re.kr (S.J.P.); kimjs@kist.re.kr (J.K.); jhwank@kist.re.kr (J.K.); dbdnjs1215@kist.re.kr (Y.K.); ehl@kist.re.kr (E.H.L.); 217014@kist.re.kr (H.J.K.); swkim24@naver.com (S.K.); t19145@kist.re.kr (B.K.); rium@kist.re.kr (R.K.); jiwon0602@kist.re.kr (J.W.C.); 2 Division of Bio-Medical Science & Technology, KIST School, Korea University of Science and Technology, Seoul 02792, Korea; 3 Department of Biotechnology, Yonsei University, Seoul 03722, Korea

**Keywords:** serinolamide A, S1P_1_ receptor, GPCR, multiple sclerosis, internalization

## Abstract

Sphingosine-1-phosphate-1 (S1P_1_) receptor agonists are well-known drugs for treating multiple sclerosis (MS) caused by autoreactive lymphocytes that attack the myelin sheath. Therefore, an effective therapeutic strategy is to reduce the lymphocytes in the blood by inducing S1P_1_ receptor internalization. We synthesized serinolamide A, a natural product of the sea, and performed S1P_1_ receptor internalization assay to evaluate functionally antagonistic S1P_1_ receptor agonist activity. In order to synthesize derivatives with better efficacy than serinolamide A and B, new derivatives were synthesized by introducing the phenyl ring moiety of fingolimod. Among them, compounds **19** and **21** had superior S1P_1_ agonistic effects to serinolamide. We also confirmed that compound **19** effectively inhibited lymphocyte outflow in peripheral lymphocyte count (PLC) assay.

## 1. Introduction

**Figure 1:**
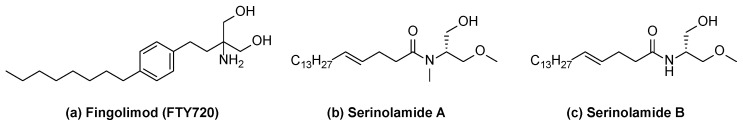
Structures of fingolimod (**a**), serinolamide A (**b**), and serinolamide B (**c**).

Multiple sclerosis (MS) is a neuroinflammatory autoimmune disease [1]. Of the different types, relapsing–remitting multiple sclerosis (RRMS) is the most common disease course [2,3]. Multiple sclerosis is caused when autoreactive T cells migrate across the blood–brain barrier (BBB) and damage the myelin sheath in the central nervous system, leading to neurodegeneration and demyelination [4]. Sphingosine-1-phosphate (S1P) receptors are a class of G protein-coupled receptors (GPCR) with five subtypes, S1P_1–5_. Among them, sphingosine-1-phosphate-1 (S1P_1_) plays a role in regulating the egress of lymphocytes from lymphoid tissue to the lymph [5]. Studies have shown that the S1P_1_ receptor is internalized and degraded by functional antagonists, prompting lymphocyte sequestration in the lymph node and immunosuppression [6,7,8]. Therefore, developing functionally antagonistic S1P_1_ receptor agonists is an effective strategy for overcoming autoimmune diseases. Natural marine products serinolamide A and serinolamide B have long lipophilic chains and polar substituents such as the well-known S1P_1_ receptor agonist fingolimod (FTY720, Gilenya^®^), as shown in Figure 1. Serinolamide A synthesis methods have been reported in multiple papers [9,10,11,12]. In this study, we partially optimized the existing synthesis method for serinolamides A and B. Serinolamide derivatives were also synthesized by introducing the phenyl moiety of FTY720. S1P_1_ receptor agonists bind to the S1P_1_ receptor, which induces S1P_1_ receptor internalization and, consequently, induces receptor degradation [5,6,7,8]. Therefore, synthesized compounds were evaluated as functional antagonists that effectively degrade S1P_1_ receptors by performing S1P_1_ receptor internalization analysis.

## 2. Results and Discussion

### 2.1. Chemical Synthesis of Serinolamide Derivatives

**Scheme 1:**
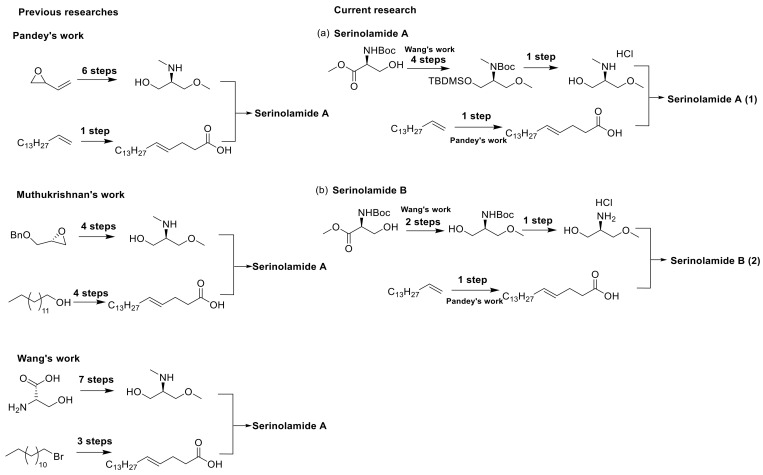
Synthesis routes for serinolamides A and B.

The synthesis of serinolamide A followed the procedures reported by Pandey [10] and Wang [11]. The amine part was synthesized following the amide coupling method reported by Wang. The *O*-methylation of commercially available methyl (*tert*-butoxycarbonyl)-*L*-serinate with commercially available methyl iodide yielded methyl *N*-(*tert*-butoxycarbonyl)-*O*-methyl-*L*-serinate **22**. The reduction of the methyl ester with sodium borohydride yielded an alcohol derivative **23**, and TBDMS protection of **23** with butyldimethylsilyl chloride (TBDMSCl) yielded **24**. The methylation of **24** with commercially available methyl iodide yielded tert-butyl (*S*)-(1-((*tert*-butyldimethylsilyl)oxy)-3-methoxypropan-2-yl)(methyl)carbamate **25**. Boc and TBDMS deprotection of **25** with 4 M HCl yielded **26**; Boc deprotection of **23** with 4 M HCl yielded **27** (Scheme S1 in the Supporting Information). The carboxylic-acid-containing counterpart was introduced following the metathesis method using the Grubbs catalyst reported by Pandey (Scheme 1) [10].

**Scheme 2:**
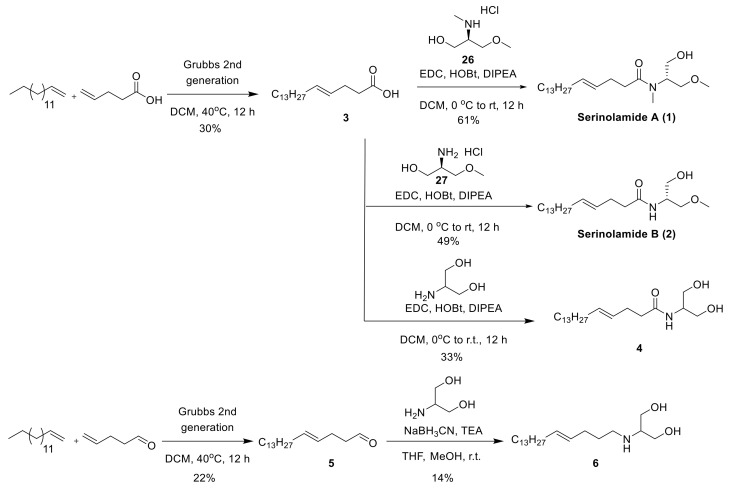
Synthesis of **1**, **2**, **4**, **6**.

The olefin metathesis reaction of commercially available pentadec-1-ene with commercially available pent-4-enoic acid yielded (*E*)-octadec-4-enoic acid **3**. Amide coupling of secondary amine derivative **26** and primary amine derivative **27** with the carboxylic acid derivative **3** yielded serinolamide A and serinolamide B (Scheme 2) [12]. Amide coupling of **3** with commercially available serinol yielded **4**. The olefin metathesis reaction of commercially available pentadec-1-ene with commercially available pent-4-enal yielded (*E*)-octadec-4-enal (**5**). The reductive amination of **5** with commercially available serinol yielded **6** (Scheme 2).

### 2.2. Chemical Synthesis of Fingolimod Analogues

**Scheme 3:**
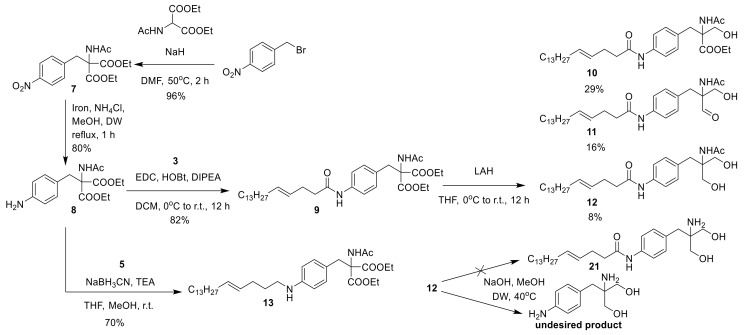
Synthesis of **10**–**13**.

The nucleophilic substitution reaction of commercially available 1-(bromomethyl)-4-nitrobenzene with commercially available diethyl 2-acetamidomalonate yielded diethyl 2-acetamido-2-(4-nitrobenzyl)malonate **7**. The reduction of the nitro group with iron yielded an amine derivative **8**, and amide coupling of **8** with carboxylic acid derivative **3** yielded **9**. Reducing the ethyl ester derivative **9** with lithium aluminum hydride (LAH) yielded compounds **10**–**12**, and the hydrolysis of **12** with sodium hydroxide yielded an undesired product (Scheme 3). The reductive amination of **8** with aldehyde derivative **5** yielded **13**.

**Scheme 4:**
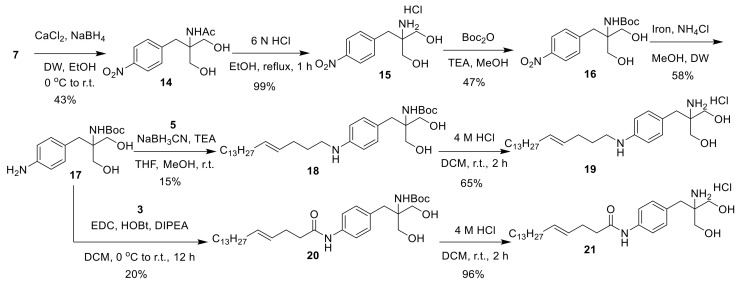
Synthesis of **19**–**21**.

Reducing ethyl ester derivative **7** with calcium chloride yielded alcohol derivative **14.** The deacetylation of **14** with 6 M HCl yielded amine salt derivative **15**. The *tert*-butyloxycarbonyl protection of amine derivative **15** with di-*tert*-butyl dicarbonate yielded compound **16**. The reduction of nitro derivative **16** with iron yielded amine derivative **17**. The reductive amination of **17** with aldehyde derivative **5** yielded compound **18**, and the Boc deprotection of **18** with 4 M HCl yielded **19**. Amide coupling of **17** with carboxylic acid derivative **3** yielded **20**, and its Boc deprotection with 4 M HCl yielded **21** (Scheme 4).

### 2.3. Evaluation of the Synthesized Serinolamide Derivatives as S1P_1_ Receptor Agonists

The ability of the compounds to internalize the S1P_1_ receptor from the cell surface was evaluated using a commercially available in vitro assay system to test the functionally antagonistic S1P_1_ receptor agonist activity of the synthesized serinolamide derivatives [13,14,15]. The efficacy of the synthetic compounds was expressed as a percentage of maximal efficacy at 1 μM of FTY720, a highly potent S1P_1_ agonist. In the S1P_1_ receptor internalization assay, compounds **12**, **19**, and **21** showed more than 80% efficacies at 30 μM. Notably, compound **19** showed good efficacy of 147%. In addition, compounds **4**, **6**, and **13** showed lower S1P_1_ receptor internalization efficacies than other derivatives (Table 1). The efficacy of compounds **19** and **21,** in which a phenyl ring was introduced, was significantly improved compared to those of compounds **4** and **6,** in which a phenyl ring was not introduced.

### 2.4. In Vivo Reduction of Peripheral Blood Lymphocyte Count by Treatment of Compounds 19 and 21 in Mice

**Figure 2:**
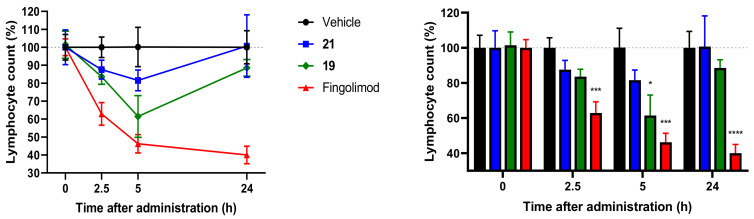
Reduction of the blood lymphocyte count by the treatment of **19** and **21** in mice. Mice were intravenously administered with the vehicle (*n* = 8), **19** (15 mg/kg, *n* = 7), **21** (30 mg/kg, *n* = 10), or positive control fingolimod (3 mg/kg, *n* = 8). Blood lymphocyte counts were measured before (0 h) and after the administration (2.5 and 5, 24 h). Percentage of the lymphocyte count at the time before administration (0 h) was considered as a baseline (100%). * *p* < 0.05, *** *p* < 0.001, **** p < 0.0001 compared to vehicle-treated group (one-way ANOVA with Dunnett’s test). Data are presented as mean ± SEM.

S1P_1_ agonists, such as fingolimod, are known to induce peripheral lymphopenia by inhibiting S1P_1_-mediated lymphocyte outflow from lymphoid tissues. Lymphopenia caused by these drugs contributes to the therapeutic effect of autoimmune diseases such as multiple sclerosis [6,16]. Therefore, we investigated the effect of compounds to induce lymphopenia in blood by peripheral lymphocyte count (PLC) analysis (Figure 2). After intravenous administration of compounds **19**, **21** (15 mg/kg, 30 mg/kg, maximum solubility concentration) and fingolimod (3 mg/kg) to mice, blood samples were collected by orbital bleed. At this time, there was no visual change compared to the vehicle treatment group containing the same amount of DMSO. As a result of measuring the number of lymphocytes in the blood, the number of lymphocytes started to decrease within 2.5 h after administration. In particular, the number of lymphocytes in the blood of mice administered **19** was significantly decreased during the first 5 h. In contrast to fingolimod, mice treated with **19** and **21** began to recover lymphocyte counts after 5 h. As a result of a single dose administration, peripheral lymphocyte counts in fingolimod-treated mice continued to decrease for 24 h post-dose. In contrast, lymphocyte counts in mice treated with compound **19** or **21** returned to near baseline levels, suggesting that the cardiac toxicity of fingolimod due to its long-term efficacy on lymphocyte reduction can be overcome (Figure 2) [17]. Collectively, these results suggest that administration of **19** and **21** can inhibit the egress of lymphocytes from lymphoid tissues to peripheral blood, and that lymphopenia can be reversed within 24 h.

## 3. Experimental Section

### 3.1. General Methods

All chemicals, reagents, and solvents were obtained from commercially available sources as reagent grades without further purification. The yields reported are for purified products and were not optimized. Synthesized compounds were checked by thin-layer chromatography (TLC) and ^1^H and ^13^C nuclear magnetic resonance (NMR), melting point (MP), high-resolution mass spectrometry (HRMS), and high-performance liquid chromatography (HPLC) analyses. Analytical thin-layer chromatography plates monitored reactions (Merck, Cat No. 1.05715, Darmstadt, Germany) and analyzed by ultraviolet light at 254 nm and 280 nm. The reactions were purified by MPLC (Biotage^®^, Isolera^™^ one, Uppsala, Sweden). The NMR spectra were recorded at 400 MHz (^1^H)/100 MHz (^13^C) using Bruker spectrometers (Billerica, USA.). Chemical shifts (*δ*) were reported in ppm downfield from tetramethylsilane (TMS). HPLC analysis was performed using a Waters E2695 system (Milford, USA.) equipped with a YMC-Triart C18 /S-5 *μ*m /12 nm/ Lot No. 17452 (150 mm × 4.6 mm diameter). The HPLC data were recorded using the following parameters: DW (0.1% AcOH)/acetonitrile. Method A: 10/90 → 100/0 in 15 min, +5 min isocratic, flow rate of 0.5 mL/min to 1.0 mL/min, λ = 254 and 280 nm. HRMS was performed with electrospray ionization on a Q-Exactive (Thermo Fisher Scientific, Waltham, USA.) instrument. Specific rotation was measured with the autopol^®^ III polarimeter (Rudolph Research Analytical, Hackettstown, NJ, USA).

### 3.2. General Procedure for Amide Coupling Reaction (Method A) 

A mixture of carboxylic acid derivatives, EDC, HOBt, and DIPEA, was dissolved in dichloromethane ([C] ~ 0.1 M) and stirred for 20 min at room temperature. Amine derivatives were added into the reaction mixture and stirred overnight to afford serinolamide A. The reaction mixture was diluted with distilled water and extracted with ethyl acetate. The combined organic layer was dried with Na_2_SO_4_ and evaporated in vacuo. The obtained residue was purified by column chromatography on SiO_2_.

### 3.3. General Procedure for Boc Deprotection and Deacetylation Reaction (Method B)

To a mixture of NHBoc derivatives in dichloromethane or ethanol ([C] ~ 0.1 M), 4 M HCl was added, and the reaction mixture was stirred at room temperature for 2 h. The reaction mixture was evaporated in vacuo.

### 3.4. General Procedure for the Reductive Amination with Aldehyde (Method C)

A mixture of aldehyde derivatives in methanol and tetrahydrofuran in a ratio of 1:1 ([C] ~ 0.1 M) was added a mixture of amine derivatives with triethylamine. The resulting suspension was stirred at room temperature (0.5 h). Then, sodium cyanoborohydride was added and stirred at room temperature. The reaction mixture was evaporated in vacuo. The product residue was washed with ethyl acetate and distilled water. The combined organic layer was dried with anhydrous Na_2_SO_4_ and evaporated in vacuo. The obtained residue was purified by column chromatography on SiO_2_.

#### 3.4.1. Synthesis of Serinolamide A (**1**)

Using Method A, **3** (50 mg, 0.17 mmol), EDC (68 mg, 0.44 mmol), HOBt (85 mg, 0.63 mmol) and DIPEA (0.23 mL, 1.33 mmol), **26** (44 mg, 0.28 mmol) gave 40 mg (61%) of serinolamide A as clear oil; *R_f_* = 0.34 (*n*-hexane/EtOAc 1/2); {[α]_D_^25^ = +2.78 (*c* = 0.18, CHCl_3_)}[Lit.^10^ [α]_D_^25^ = +1.97 (*c* = 0.18, CHCl_3_)]; IR (KBr): *ν* 3361, 2955, 2917, 2848, 1732, 1616, 1469 cm^−1^; ^1^H NMR (CDCl_3_, 400 MHz) *δ* 5.43–5.47 (m, trans–2H), 4.37–4.40 (m, 1H), 3.54–3.78 (m, 5H), 3.47 (s, 3H), 3.01–2.83 (m, 3H), 2.30–2.43 (m, 4H), 1.95–1.98 (m, 2H), 1.24–1.32 (m, 22H), 0.88 (t, *J* = 7.0 Hz, CH_3_); ^13^C NMR (CDCl_3_,100 MHz) *δ* 174.3, 131.6, 128.4, 70.9, 62.0, 58.9, 57.4, 34.2, 33.5, 32.5, 31.9, 29.6, 29.6, 29.5, 29.3, 29.2, 28.0, 22.6, 14.1; HRMS (M + H)^+^ (ESI^+^) 384.3478 [M + H]^+^ (calcd for C_23_H_45_NO_3_H^+^ 384.3477).

#### 3.4.2. Synthesis of Serinolamide B (**2**)

Using Method A, **3** (120 mg, 0.42 mmol), EDC (169 mg, 1.09 mmol), HOBt (212 mg, 1.15 mmol) and DIPEA (0.6 mL, 3.29 mmol), **27** (96 mg, 0.68 mmol) gave 45 mg (49%) of serinolamide B as clear oil; *R_f_* = 0.13 (*n*-hexane/EtOAc 1/1); mp: 84–86 °C; [α]_D_^25^ = −17.22 (*c* = 0.18, CHCl_3_); IR (KBr): *ν* 3291, 2917, 2849, 1639, 1543, 1466 cm^−1^; ^1^H NMR (CDCl_3_, 400 MHz) *δ* 6.19–6.21 (m, NH), 5.37–5.48 (m, trans–2H), 4.02–4.07 (m, 1H), 3.75–3.78 (m, 1H), 3.61–3.65 (m, 1H), 3.54–3.58 (m, 1H), 3.47–3.51 (m, 1H), 3.34 (s, OCH_3_), 3.16–3.19 (m, 1H), 2.24–2.31 (m, 4H), 1.92–1.97 (m, 2H), 1.23–1.30 (m, 22H), 0.86 (t, *J* = 7.0 Hz, CH_3_); ^13^C NMR (CDCl_3_, 100 MHz) *δ* 173.1, 132.1, 127.9, 72.9, 63.8, 59.2, 50.5, 36.6, 32.5, 31.9, 29.6, 29.6, 29.5, 29.4, 29.3, 29.2, 28.6, 22.6, 14.1; HRMS (M + H)^+^ (ESI^+^) 370.3321 [M + H]^+^ (calcd for C_22_H_43_NO_3_H^+^ 370.3321).

#### 3.4.3. Synthesis of (E)-*N*-(1,3-dihydroxypropan-2-yl)octadec-4-enamide (**4**)

Using Method A, **3** (180 mg, 0.63 mmol), EDC (254 mg, 1.64 mmol), HOBt (122 mg, 0.90 mmol) and DIPEA (0.86 mL, 4.93 mmol), commercially available serinol (158 mg, 1.01 mmol) gave 80 mg (33%) of **4** as a white solid; *R_f_* = 0.1 (*n*-hexane/EtOAc 1/1); mp: 119–121 °C; IR (KBr): *ν* 3285, 2955, 2917, 2849, 1637, 1545, 1465 cm^−1^; ^1^H NMR (CDCl_3_, 400 MHz) *δ* 6.19 (br, 1H), 5.36–5.53 (m, trans–2H), 3.88–3.97 (m, 1H), 3.83–3.86 (m, 2H), 3.76–3.81 (m, 2H), 2.40–2.42 (m, 2H), 2.27–2.34 (m, 4H), 1.94–1.99 (m, 2H), 1.23–1.33 (m, 22H), 0.87 (t, *J* = 7.0 Hz, CH_3_); ^13^C NMR (DMSO-*d*_6_, 100 MHz) *δ* 171.9, 130.7, 129.4, 60.6, 53.2, 35.8, 32.4, 31.7, 29.5, 29.4, 29.4, 29.3, 29.1, 29.0, 28.7, 22.5, 14.4; HRMS (M + H)^+^ (ESI^+^) 356.3165 [M + H]^+^ (calcd for C_21_H_41_NO_3_H^+^ 356.3164).

#### 3.4.4. Synthesis of (E)-2-(octadec-4-en-1-ylamino)propane-1,3-diol (**6**)

Using Method C, **5** (100 mg, 0.37 mmol), commercially available serinol (38 mg, 0.41 mmol), triethylamine (0.15 mL, 1.11 mmol) and sodium cyanoborohydride (46.5 mg, 0.74 mmol) gave 18 mg (14%) of **6** as yellow oil; *R_f_* = 0.1 (*n*-hexane/EtOAc 1/1); IR (KBr): *ν* 3291, 2918, 2850, 1636, 1389, 1358 cm^−1^; ^1^H NMR (CD_3_OD, 400 MHz) *δ* 5.41–5.59 (m, trans–2H), 3.74–3.86 (m, 4H), 3.25–3.29 (m, 1H), 3.09–3.13 (m, 2H), 2.12–2.17 (m, 2H), 1.98–2.06 (m, 2H), 1.78–1.84 (m, 2H), 1.24–1.32 (m, 22H), 0.88 (t, *J* = 7.0 Hz, CH_3_); ^13^C NMR (CD_3_OD, 100 MHz) *δ* 132.1, 127.7, 60.3, 57.5, 44.6, 32.1, 31.6, 29.3, 29.3, 29.2, 29.2, 29.1, 29.0, 28.8, 25.6; HRMS (M + H)^+^ (ESI^+^) 342.3372 [M + H]^+^ (calcd for C_21_H_43_NO_2_H^+^ 342.3372).

#### 3.4.5. Synthesis of diethyl (E)-2-acetamido-2-(4-(octadec-4-enamido)benzyl)malonate (**9**)

Using Method A, **3** (105 mg, 0.37 mmol), EDC (77 mg, 0.49 mmol), HOBt (38 mg, 0.27 mmol) and DIPEA (0.26 mL, 1.5 mmol), **8** (100 mg, 0.3 mmol) gave 150 mg (82%) of **9** as clear oil; *R_f_* = 0.53 (*n*-hexane/EtOAc 1/1); IR (KBr): *ν* 3235, 2848, 1742, 1707, 1512 cm^−1^; ^1^H NMR (CDCl_3_, 400 MHz) *δ* 7.40 (d, *J* = 8.2 Hz, 2 ArH), 7.22 (s, CONH), 6.94 (d, *J* = 8.3 Hz, 2 ArH), 6.52 (s, NH), 5.42–5.57 (m, trans–2H), 4.22–4.29 (m, 1H), 3.60 (s, CH_2_), 2.40 (br, 4H), 2.02 (s, COCH_3_), 1.96–1.99 (m, 2H), 1.25–1.30 (m, 22H), 0.87 (t, *J* = 7.0 Hz, CH_3_); ^13^C NMR (CDCl_3_, 100 MHz) *δ* 169.0, 167.4, 137.0, 132.5, 130.9, 130.4, 128.0, 119.5, 67.2, 62.6, 37.6, 37.2, 32.5, 31.9, 29.6, 29.6, 29.5, 29.4, 29.3, 29.1, 28.4, 23.0, 22.6, 14.1, 14.0; HPLC purity: 15.2 min, 100%; HRMS (M + H)^+^ (ESI^+^) 587.4060 [M + H]^+^ (calcd for C_34_H_54_N_2_O_6_H^+^ 587.4060).

#### 3.4.6. Synthesis of **10**, **11**, **12**

A mixture of **9** (110 mg, 0.19 mmol) in tetrahydrofuran (5 mL) was added lithium aluminum hydride (36 mg, 0.94 mmol) at 0 ^o^C for 5 min. The resulting suspension was stirred at room temperature for 12 h. The mixture was filtered through a pad of celite and the solvent was evaporated. The residue was purified by column chromatography to give **10** (30 mg, 29%, white solid, *R_f_* = 0.5); mp: 69–71 °C, **11** (15 mg, 16%, ivory solid, *R_f_* = 0.33); mp: 84–86 °C, **12** (8 mg, 8%, ivory solid, *R_f_* = 0.16) (*n*-hexane/EtOAc 1/5); mp: 130–132 °C; **10** IR (KBr): *ν* 3158, 2845, 2765, 1731, 1698, 1478 cm^−1^; ^1^H NMR (CDCl_3_, 400 MHz) *δ* 7.42 (d, *J* = 8.4 Hz, 2 ArH), 7.13 (s, CONH), 7.05 (d, *J* = 8.4 Hz, 2 ArH), 5.86–5.88 (m, NH), 5.43–5.58 (m, trans–2H), 4.81–4.86 (m, 1H), 4.14–4.20 (m, CH_2_), 3.05–3.14 (m, 2H), 2.41 (m, 4H), 1.98–1.99 (m, 5H), 1.24–1.31 (m, 28H), 0.87 (t, *J* = 7.0 Hz, CH_3_); ^13^C NMR (CDCl_3_, 100 MHz) *δ* 171.5, 170.7, 169.5, 136.9, 132.5, 131.6, 129.8, 128.0, 119.8, 61.5, 53.1, 37.6, 37.3, 32.5, 31.9, 29.6, 29.6, 29.6, 29.5, 29.4, 29.3, 29.1, 28.4, 23.2, 22.6, 14.1; HPLC purity: 15.2 min, 94.5%; HRMS (M + H)^+^ (ESI^+^) 545.3954 [M + H]^+^ (calcd for C_32_H_52_N_2_O_5_H^+^ 545.3954). **11** IR (KBr): *ν* 3234, 2954, 2914, 2848, 1742, 1707, 1511, 1470 cm^−1^; ^1^H NMR (CDCl_3_, 400 MHz) *δ* 9.62 (s, CHO), 7.43 (d, *J* = 8.2 Hz, 2 ArH), 7.17 (s, CONH), 7.09 (d, *J* = 8.3 Hz, 2 ArH), 5.94–5.96 (m, NH), 5.43–5.56 (m, trans–2H), 4.67–4.72 (m, 1H), 3.11–3.15 (m, 2H), 2.40–2.41 (m, 4H), 1.96–2.01 (m, 5H), 1.25–1.31 (m, 25H), 0.87 (t, *J* = 7.0 Hz, CH_3_); ^13^C NMR (CDCl_3_, 100 MHz) *δ* 198.7, 170.8, 170.0, 136.9, 132.6, 131.2, 129.8, 127.9, 120.1, 59.8, 37.6, 34.4, 32.5, 31.9, 29.6, 29.6, 29.5, 29.4, 29.3, 29.1, 28.4, 23.0, 22.6, 14.1; HPLC purity: 7.7 min, 99.0%; HRMS (M + H)^+^ (ESI^+^) 501.3692 [M + H]^+^ (calcd for C_30_H_48_N_2_O_4_H^+^ 501.3692). **12** IR (KBr): *ν* 3342, 2953, 2914, 2849, 1686, 1515, 1470 cm^−1^; ^1^H NMR (CD_3_OD, 400 MHz) *δ* 7.45–7.47 (m, 2 ArH), 7.17–7.19 (m, 2 ArH), 5.44–5.55 (m, trans–2H), 3.58–3.69 (m, 4H), 3.02 (s, CH_2_), 2.37–2.41 (m, 4H), 1.99–2.00 (m, 2H), 1.96 (s, COCH_3_), 1.26–1.36 (m, 22H), 0.91 (t, *J* = 7.0 Hz, CH_3_); ^13^C NMR (CD_3_OD, 100 MHz) *δ* 172.7, 172.5, 136.8, 132.5, 131.5, 130.5, 128.0, 119.8, 119.6, 61.8, 61.7, 36.6, 36.0, 34.2, 32.1, 31.6, 29.3, 29.3, 29.2, 29.0, 28.7, 28.4, 22.3, 13.0; HPLC purity: 14.0 min, 98.6%; HRMS (M + H)^+^ (ESI^+^) 503.3849 [M + H]^+^ (calcd for C_30_H_50_N_2_O_4_H^+^ 503.3848).

#### 3.4.7. Synthesis of diethyl (E)-2-acetamido-2-(4-(octadec-4-en-1-ylamino)benzyl)malonate (**13**)

Using Method C, **5** (165 mg, 0.62 mmol), **8** (200 mg, 0.62 mmol), triethylamine (0.26 mL, 1.86 mmol) and sodium cyanoborohydride (78 mg, 1.24 mmol) gave 150 mg (70%) of **13** as a white solid; *R_f_* = 0.3 (*n*-hexane/EtOAc 1/2); mp: 67–69 °C; IR (KBr): *ν* 3205, 2946, 2814, 1673, 1480 cm^−1^; ^1^H NMR (CDCl_3_, 400 MHz) *δ* 6.79 (d, *J* = 8.3 Hz, 2 ArH), 6.52 (s, CONH), 6.47 (d, *J* = 8.4 Hz, 2 ArH), 5.41–5.43 (m, trans–2H), 4.22–4.28 (m, 4H), 3.56–3.63 (m, 1H), 3.51 (s, 2H), 3.05–3.08 (m, 2H), 1.96–2.09 (m, 7H), 1.63–1.67 (m, 2H), 1.25–1.30 (m, 28H), 0.87 (t, *J* = 7.0 Hz, CH_3_); ^13^C NMR (CDCl_3_, 100 MHz) *δ* 168.9, 167.7, 147.5, 131.4, 130.6, 129.1, 123.2, 122.5, 67.4, 62.4, 43.4, 37.0, 32.5, 31.9, 30.1, 29.6, 29.6, 29.5, 29.5, 29.3, 29.2, 23.0, 22.6, 14.1, 14.0; HPLC purity: 17.5 min, 95.5%; HRMS (M + H)^+^ (ESI^+^) 573.4268 [M + H]^+^ (calcd for C_34_H_56_N_2_O_5_H^+^ 573.4267).

#### 3.4.8. Synthesis of (E)-2-amino-2-(4-(octadec-4-en-1-ylamino)benzyl)propane-1,3-diol (**19**)

Using Method B, **18** (70 mg, 0.12 mmol) and 4 M HCl in dioxane (0.16 mL, 0.64 mmol) gave 37 mg (65%) of **19** as a brown solid; mp: 135–137 °C (decomp.); [α]_D_^25^ = +1.11 (*c* = 0.18, EtOH); IR (KBr): *ν* 3301, 2919, 2850, 1664, 1513, 1412, 1163 cm^−1^; ^1^H NMR (CD_3_OD, 400 MHz) *δ* 7.57 (br, 4 ArH), 5.39–5.55 (m, trans–2H), 3.51–3.58 (m, 4H), 3.32–3.39 (m, 2H), 3.13 (s, CH**_2_**), 2.13–2.18 (m, 2H), 1.98–2.03 (m, 2H), 1.86 (br, 2H), 1.26–1.37 (m, 22H), 0.91 (t, *J* = 6.9 Hz, CH_3_); ^13^C NMR (CD_3_OD, 100 MHz) *δ* 136.4, 134.5, 132.3, 132.1, 127.6, 122.8, 61.0, 60.2, 51.7, 35.3, 32.2, 31.6, 29.4, 29.3, 29.2, 29.1, 28.9, 28.9, 25.5, 22.3, 13.1; HPLC purity: 7.8 min, 96.1%; HRMS (M + H)^+^ (ESI^+^) 447.3951 [M + H]^+^ (calcd for C_28_H_50_N_2_O_2_H^+^ 447.3950).

#### 3.4.9. Synthesis of (E)-*N*-(4-(2-amino-3-hydroxy-2-(hydroxymethyl)propyl)phenyl)octadec-4-enamide (**21**)

Using Method B, **20** (12 mg, 0.02 mmol) and 4 M HCl in dioxane (0.02 mL, 0.08 mmol) gave 10 mg (96%) of **21** as an ivory solid; mp: 156–158 °C (decomp.); [α]_D_^25^ = +7.22 (*c* = 0.18, EtOH); IR (KBr): *ν* 3288, 2916, 2849, 1655, 1598, 1511, 1414, 1119, 1050 cm^−1^; ^1^H NMR (CD_3_OD, 400 MHz) *δ* 7.54 (d, *J* = 8.3 Hz, 2 ArH), 7.27 (d, *J* = 8.3 Hz, 2 ArH), 5.43–5.56 (m, trans–2H), 3.55 (s, 4H), 2.99 (s, CH_2_), 2.38–2.43 (m, 4H), 1.98–2.03 (m, 2H), 1.27–1.34 (m, 22H), 0.92 (t, *J* = 7.0 Hz, CH_3_); ^13^C NMR (CD_3_OD, 100 MHz) *δ* 131.5, 130.5, 129.6, 128.0, 120.1, 61.0, 60.4, 36.6, 35.4, 32.1, 31.6, 29.3, 29.3, 29.1, 29.0, 28.7, 28.4, 22.3, 13.0; HPLC purity: 8.8 min, 99.1%; HRMS (M + H)^+^ (ESI^+^) 461.3743 [M + H]^+^ (calcd for C_28_H_48_N_2_O_3_H^+^ 461.3743).

### 3.5. Cell Culture

For S1P_1_ receptor internalization assay, PathHunter^®^ EDG1 HEK 293 cells (93-0784C1; DiscoverX, Fremont, CA, USA.) were cultured in DMEM containing 10% (*v*/*v*) fetal bovine serum (Biowest), 100 U/mL penicillin-streptomycin (Gibco), 0.25 μg/mL puromycin (Invivogen), and 200 μg/mL hygromycin B (Invitrogen). Cells were incubated at 5% CO_2_ in a 37 °C humidified atmosphere.

### 3.6. S1P_1_ Receptor Internalization Assay

The S1P_1_ receptor internalization activity of synthesized compounds was evaluated using PathHunter^®^ EDG1 total GPCR internalization HEK293 cell line (93-0784C1; DiscoverX). The cell lines are engineered to co-express two fragments of *β*-galactosidase at S1P_1_ receptor and endosome, respectively. The endocytosis of receptor leads *β*-galactosidase to complemented form, and the internalization activity was measured by chemiluminescent signal. The HEK293 EDG1 cells (1 × 10^4^ cells/well) in cell plating 28 reagent (DiscoverX) were seeded in 96-well white plates and incubated overnight at 37 °C. The test compounds were prepared in cell plating 28 Reagent (DiscoverX) and treated for 3 h at 37 °C. Then, 50 μL of detection reagent (PathHunter^®^ Detection Kit, 93-0001L; DiscoverX) was added to the wells and incubated for 1 h at room temperature in the dark. The chemiluminescent signals were measured at all wavelengths using a microplate reader (SpectraMax^®^*i*3; Molecular Devices).

### 3.7. Measurement of Peripheral Lymphocyte Count

Compound **19** and fingolimod were dissolved in 5% DMSO and distilled water, and **21** was dissolved in 50% DMSO and distilled water (final volume was 20 μL). All test compounds were intravenously administered to B6C3H mice (10 wks, 20 g). Blood samples were obtained from retro-orbital sinus of the mice under anesthesia (4% isoflurane) at different time points and were collected into a K2-EDTA-coated tube. Blood lymphocyte counts were measured using an automatic blood cell counter (Horiba).

## 4. Conclusions

In this study, we optimized previous synthetic methods for serinolamides A and B. Structural similarities with FTY720 indicated that serinolamides may act as S1P_1_ receptor agonists. We synthesized a series of derivatives and evaluated their efficacy in S1P_1_ receptor internalization. Compounds **19** and **21** were rationally designed by hybridization of serinolamide A with the FTY720 scaffold and exhibited favorable efficacies in S1P_1_ receptor internalization. Finally, we confirmed that compound **19** in vivo activity by effectively reducing the number of blood lymphocytes in mice.

## Figures and Tables

**Table 1:** Effects of Serinolamide A derivatives on ligand binding to G protein-coupled receptor.

Compounds	S1P_1_ Receptor Internalization ^1^		
	30 μM (% Efficacy ^2^)	20 μM (% Efficacy ^2^)	10 μM (% Efficacy ^2^)
**1** **(Serinolamide A)**	60.4	36.2	30.1
**2** **(Serinolamide B)**	53.5	46.4	31.7
**4**	27.8	22.5	15.8
**6**	0.98	13.7	nd ^3^
**9**	44.4	40.1	nd ^3^
**10**	41.3	41.2	nd ^3^
**11**	69.0	78.0	nd ^3^
**12**	84.4	77.8	43.1
**13**	22.2	16.7	nd ^3^
**19**	147	107.3	37.8
**21**	94.2	97.5	34.2

^1^ The activity of S1P_1_ receptor internalization was determined based on HEK293-S1P_1_ expressing cells. ^2^ % efficacy was calculated compared to 1 μM of FTY720-phosphate, the active pharmacological species. ^3^ nd = not determined.

## Data Availability

Data are contained within the article or the Supplementary Materials file.

## Supplementary Materials

The following supporting information can be downloaded at: https://www.mdpi.com/article/10.3390/molecules27092818/s1, Scheme S1: Synthesis of **26** and **27**.

## Sample Availability

Samples of the compounds are available from the authors upon the reasonable request.
