# Supplementary material for: Synthesis and Evaluation of Serinolamide Derivatives as Sphingosine-1-Phosphate-1 (S1P1) Receptor Agonists
Source: Molecules. 2022 Apr 28;27(9):2818. doi: 10.3390/molecules27092818 (PMC9101607; doi:10.3390/molecules27092818)
Supplement: Supplementary file 1 [file molecules-27-02818-s001.zip › molecules-1690130-supplementary.pdf]

molecules MDPI

Supplementary Materials

Synthesis and Evaluation of Serinolamide Derivatives as Sphingosine-1-Phosphate-1 ($S1P_1$) Receptor Agonists

Sun Jun Park [1,2,†], Jushin Kim [1,3,†], Jaehwan Kim [1,2], Yoowon Kim [1,3], Elijah Hwejin Lee [1,2], Hyeon Jeong Kim [1,3], Siwon Kim [1], Byungeun Kim [1,2], Rium Kim [1,2], Ji Won Choi [1], Jong-Hyun Park [1,2,*] and Ki Duk Park [1,2,*]

[1] Convergence Research Center for Brain Science, Korea Institute of Science & Technology (KIST), Seoul 02792, Korea; h18502@kist.re.kr (S.J.P.); kimjs@kist.re.kr (J.K.); jhwank@kist.re.kr (J.K.); dbdnjs1215@kist.re.kr (Y.K.); ehl@kist.re.kr (E.H.L.); 217014@kist.re.kr (H.J.K.); swkim24@naver.com (S.K.); t19145@kist.re.kr (B.K.); rium@kist.re.kr (R.K.); jiwon0602@kist.re.kr (J.W.C.)

[2] Division of Bio-Medical Science & Technology, KIST School, Korea University of Science and Technology, Seoul 02792, Korea

[3] Department of Biotechnology, Yonsei University, Seoul 03722, Korea

* Correspondence: jhyunprk@kist.re.kr (J.-H.P.); kdpark@kist.re.kr (K.D.P.)

† These authors contributed equally to this work.

Table of Contents:

1. Experimental section -- S2

General Methods --- S2

2. Supplemental Experimental Section ---S3

Scheme S1. Synthesis of 26 and 27--- S6

3. ^{1}H NMR and ^{13}C NMR spectra for the final compounds --------------------------------------- S8

4. HPLC analysis for the final compounds -- S19

5. HRMS analysis for the final compounds --- S23

6. Reference --- S29

1. Experimental section

General Methods

All chemicals, reagents, and solvents were obtained from commercially available sources as reagent grades without further purification. Yields reported are for purified products and were not optimized. Synthesized compounds were checked by thin-layer chromatography (TLC) and ^{1}H and ^{13}C nuclear magnetic resonance (NMR), melting point (MP), high-resolution mass spectrometry (HRMS), and high-performance liquid chromatography (HPLC) analyses. Analytical thin-layer chromatography plates monitored reactions (Merck, Cat No. 1.05715, Darmstadt, Germany) and analyzed by ultraviolet light at 254 nm and 280 nm. The reactions were purified by MPLC (Biotage®, Isolera™ one, Uppsala, Sweden). The NMR spectra were recorded at 400 MHz (^{1}H)/100 MHz (^{13}C) or 300 MHz (^{1}H)/75 MHz (^{13}C) using Bruker spectrometers (Billerica, USA.). Chemical shifts (δ) were reported in ppm downfield from tetramethylsilane (TMS). HPLC analysis was performed using a Waters E2695 system (Milford, USA.) equipped with a YMC-Triart C18 /S-5 μm /12 nm/ Lot No. 17452 (150 mm × 4.6 mm diameter). The HPLC data were recorded using the following parameters: DW (0.1% AcOH)/acetonitrile. Method A: 10/90 → 100/0 in 15 min, +5 min isocratic, flow rate of 0.5 mL/min to 1.0 mL/min, λ = 254 and 280 nm. HRMS was performed with electrospray ionization on a Q-Exactive (Thermo Fisher Scientific, Waltham, U.S.A.) instrument. Specific rotation was measured with the autopol® III polarimeter (Rudolph Research Analytical, Hackettstown, USA.).

2. Supplemental Experimental Section

General procedure for amide coupling reaction (Method A).
To a mixture of carboxylic acid derivatives (1.0 eq), EDC (2.6 eq), HOBt (1.4 eq) and DIPEA (7.84 eq) were dissolved in dichloromethane ([C] ~ 0.1 M) and stirred for 20 min at room temperature. Amine derivatives (1.6eq) was added into the reaction mixture and stirred overnight to afford serinolamide A. The reaction mixture was diluted with distilled water and extracted with ethyl acetate. The combined organic layer was dried with Na_2SO_4 and evaporated in vacuo. The obtained residue was purified by column chromatography on SiO_2.

General procedure for boc deprotection and deacetylation reaction (Method B).
To a mixture of NHBoc derivatives (1.0 eq) in dichloromethane or ethanol ([C] ~ 0.1 M), 4~6 M HCl (5.0 eq) was added and reaction mixture was stirred at room temperature for 2 h. The reaction mixture was evaporated in vacuo.

General procedure for the reductive amination with aldehyde (Method C).
To a mixture of aldehyde derivatives in methanol and tetrahydrofuran in a ratio of 1:1 ([C] ~ 0.1 M) was added a mixture of amine derivatives (1.5 eq) with triethylamine (3.0 eq). The resulting suspension was stirred at room temperature (0.5 h). Then, sodium cyanoborohydride (4.0 eq) was added and stirred at room temperature (2–3 h). The reaction mixture was evaporated in vacuo. The product residue was washed with ethyl acetate and distilled water. The combined organic layer was dried with anhydrous Na_2SO_4 and evaporated in vacuo. The obtained residue was purified by column chromatography on SiO_2.

Synthesis of (*E*)-octadec-4-enoic acid (3)

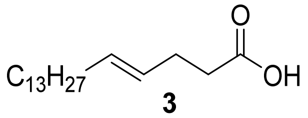

3

To a mixture of commercially available 1-pentadecene (2 mL, 9.50 mmol) in dichloromethane (20 mL) was added commercially available 4-pentenoic acid (0.97 mL, 9.50 mmol) then was added Grubbs 2nd generation catalyst (59 mg, 0.24 mmol). The resulting suspension was stirred at 40 °C for 24 h. The mixture was filtered through a pad of celite and the solvent was evaporated. The residue was purified by column chromatography to give **3** (800 mg, 30%) as a white solid; R_f = 0.51 (*n*-hexane/EtOAc 2/1); ^1H NMR ($CDCl_3$, 400 MHz) δ 5.36–5.52 (m, trans–2H), 2.40–2.44 (m, 2H), 2.29–2.34 (m, 2H), 1.94–1.99 (m, 2H), 1.25–1.30 (m, 22H), 0.88 (t, *J* = 7.0 Hz, CH_3); ^{13}C NMR ($CDCl_3$, 100 MHz) δ 179.3, 132.2, 127.4, 34.1, 32.5, 31.9, 29.7, 29.5, 29.4, 29.3, 29.1, 27.5, 22.7, 14.1.

Synthesis of (*E*)-octadec-4-enal (5)

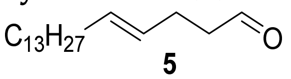

5

To a mixture of commercially available 1-pentadecene (2 mL, 9.5 mmol) in dichloromethane (20 mL) was added commercially available 4-pentenal (1 mL, 9.5 mmol) then was added Grubbs 2nd generation catalyst (201 mg, 0.24 mmol). The resulting suspension was stirred at 40 °C for 24 h. The mixture was filtered through a pad of celite and the solvent was evaporated. The residue was purified by column chromatography to give **5** (550 mg, 22%) as a white solid; R_f = 0.50 (*n*-hexane/EtOAc 10/1); ^{1}H NMR ($CDCl_3$, 400 MHz) δ 9.97–9.75 (m, CHO), 5.34–5.49 (m, trans–2H), 2.45–2.49 (m, 2H), 2.29–2.34 (m, 2H), 1.93–1.98 (m, 2H), 1.24–1.33 (m, 22H), 0.87 (t, J = 7.0 Hz, CH_3); ^{13}C NMR ($CDCl_3$, 100 MHz) δ 202.3, 132.1, 127.5, 43.5, 32.4, 31.9, 29.6, 29.6, 29.4, 29.4, 29.3, 29.1, 25.1, 22.6, 14.0.

Synthesis of diethyl 2-acetamido-2-(4-nitrobenzyl)malonate (7) [1]

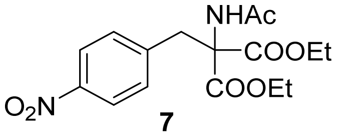

7

To a mixture of commercially available diethyl 2-acetamidomalonate (2 g, 9.25 mmol) in anhydrous *N*,*N*-dimethylformamide (20 mL) was slowly added 60% sodium hydride (370 mg, 9.25 mmol) at 0 °C then was added commercially available 1-(bromomethyl)-4-nitrobenzene (2 g, 9.25 mmol). The resulting suspension was stirred at 50 °C (2 h). The reaction mixture was diluted with distilled water and extracted with ethyl acetate. The combined organic layer was dried with anhydrous Na_2SO_4. The residue was purified by column chromatography to give **7** (1.8 g, 96%) as clear oil; R_f = 0.4 (*n*-hexane/EtOAc 1/1); ^{1}H NMR ($CDCl_3$, 400 MHz) δ 8.12–8.14 (m, 2 ArH), 7.18 (d, J = 8.7 Hz, 2 ArH), 6.53 (s, NH), 4.25–4.32 (m, 4H), 3.78 (s, CH_2), 2.04 (s, $COCH_3$), 1.28–1.32 (m, 6H).

Synthesis of diethyl 2-acetamido-2-(4-aminobenzyl)malonate (8) [1]

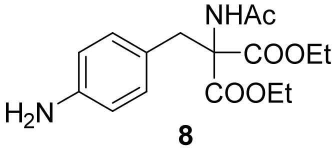

8

To a mixture of **7** (1.1 g, 3.12 mmol) in methanol (20 mL) and DW (20 mL) was added NH_4Cl (1.7 g, 31.2 mmol) and Iron (871 mg, 15.6 mmol). The resulting suspension was stirred at 80 °C (1 h). The product mixture was filtered and evaporated in vacuo. The residue was purified by column chromatography to give **8** (800 mg, 80%) as a ivory solid; R_f = 0.2 (*n*-hexane/EtOAc 1/1); ^{1}H NMR ($CDCl_3$, 400 MHz) δ 6.78 (d, J = 8.4 Hz, 2 ArH), 6.56–6.58 (m, 2 ArH), 6.51 (s, NH), 4.22–4.28 (m, 4H), 3.59 (s, NH_2), 3.52 (s, CH_2), 2.02 (s, $COCH_3$), 1.27–1.30 (m, 6H).

Synthesis of *N*-(1,3-dihydroxy-2-(4-nitrobenzyl)propan-2-yl)acetamide (14)

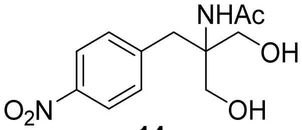

14

To a mixture of **7** (600 mg, 1.7 mmol) in ethanol (5 mL) and DW (15 mL) was added $CaCl_2$ (1.2 g, 8.5 mmol) at 0 °C then was slowly added $NaBH_4$ (514 mg, 13.6 mmol) at 0 °C. The resulting suspension was stirred at room temperature (12 h). The reaction mixture was diluted with distilled water and extracted with ethyl acetate. The combined organic layer was dried with anhydrous Na_2SO_4. The residue was purified by column chromatography to give **14** (650 mg, 43%) as a white solid; ^1H NMR (CD_3OD, 400 MHz) δ 8.17 (d, J = 8.7 Hz, 2 ArH), 7.48 (d, J = 8.7 Hz, 2 ArH), 3.72 (d, J = 11.0 Hz, CH_2OH), 3.55 (d, J = 11.0 Hz, CH_2OH), 3.21 (s, CH_2), 1.96 (s, $COCH_3$).

Synthesis of 2-amino-2-(4-nitrobenzyl)propane-1,3-diol hydrochloride (15)

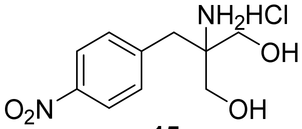

15

Using Method B, **14** (300 mg, 1.11 mmol) and aqueous 6 N HCl (5 mL) gave 290 mg (99%) of **15** as a ivory solid; ^1H NMR (CD_3OD, 400 MHz) δ 8.23–8.25 (m, 2 ArH), 7.60 (d, J = 8.6 Hz, 2 ArH), 3.50–3.58 (m, 4H), 3.18 (s, CH_2).

Synthesis of *tert*-butyl (1,3-dihydroxy-2-(4-nitrobenzyl)propan-2-yl)carbamate (16)

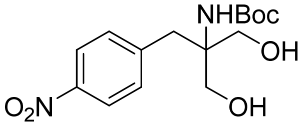

16

To a mixture of **15** (290 mg, 1.1 mmol) in methanol (10 mL) was added triethylamine (0.23 mL, 1.65 mmol) then was added Boc_2O (360 mg, 1.65 mmol). The resulting suspension was stirred at 60 °C (12 h). The reaction mixture was diluted with distilled water and extracted with ethyl acetate. The combined organic layer was dried with anhydrous Na_2SO_4. The residue was purified by column chromatography to give **16** (170 mg, 47%) as clear oil; R_f = 0.66 (EtOAc 100%); ^1H NMR (CD_3OD, 400 MHz) δ 8.15 (d, J = 8.7 Hz, 2 ArH), 7.49 (d, J = 8.7 Hz, 2 ArH), 3.65 (d, J = 10.9 Hz, CH_2OH), 3.56 (d, J = 10.9 Hz, CH_2OH), 3.17 (s, CH_2), 1.49 (s, $C(CH_3)_3$.

Synthesis of *tert*-butyl (2-(4-aminobenzyl)-1,3-dihydroxypropan-2-yl)carbamate (17)

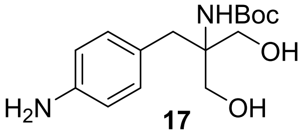

17

To a mixture of **16** (510 mg, 1.56 mmol) in methanol (20 mL) and DW (20 mL) was added NH_4Cl (836 mg, 15.6 mmol) and Iron (436 mg, 7.8 mmol). The resulting suspension was stirred at 80 °C (1 h). The product mixture was filtered and evaporated in vacuo. The

residue was purified by column chromatography to give **17** (270 mg, 58%) as a ivory solid; R_f = 0.43 (*n*-hexane/EtOAc 1/5); ^{1}H NMR (CD_3OD, 400 MHz) δ 6.99 (d, *J* = 8.2 Hz, 2 ArH), 6.67 (d, *J* = 8.3 Hz, 2 ArH), 3.55–3.63 (m, 4H), 2.89 (s, CH_2), 1.48 (s, $C(CH_3)_3$.

Synthesis of *tert*-butyl (*E*)-(1,3-dihydroxy-2-(4-(octadec-4-en-1-ylamino)benzyl)propan-2-yl)carbamate (18)

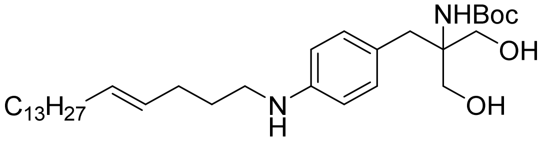

18

Using Method C, **5** (234 mg, 0.87 mmol), **17** (260 mg, 0.87 mmol), triethylamine (0.36 mL, 2.61 mmol) and sodium cyanoborohydride (109 mg, 1.74 mmol) gave 70 mg (15%) of **18** as ivory oil; R_f = 0.71 (*n*-hexane/EtOAc 2/1); ^{1}H NMR ($CDCl_3$, 400 MHz) δ 7.01 (d, *J* = 8.2 Hz, 2 ArH), 6.56 (d, *J* = 8.2 Hz, 2 ArH), 5.42–5.44 (m, trans–2H), 4.89 (s, 1H), 4.33–4.46 (m, 1H), 3.76 (d, *J* =11.5, CH_2OH), 3.54 (d, *J* =11.6, CH_2OH), 3.08–3.12 (m, 2H), 1.95–2.18 (m, 4H), 1.65–1.70 (m, 2H), 1.42 (s, $C(CH_3)_3$), 1.24–1.34 (m, 28H), 0.88 (t, *J* = 7.0 Hz, CH_3).

Synthesis of tert-butyl (*E*)-(1,3-dihydroxy-2-(4-(octadec-4-enamido)benzyl)propan-2-yl)carbamate (20)

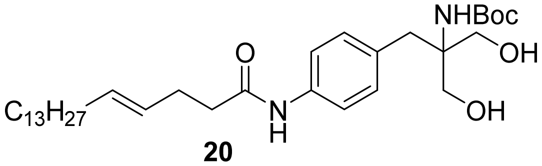

20

Using Method A, **3** (34 mg, 0.12 mmol), EDC (25 mg, 0.16 mmol), HOBt (12 mg, 0.09 mmol) and DIPEA (0.85 mL, 0.49 mmol), **17** (30 mg, 0.10 mmol) gave 12 mg (20%) of **20** as clear oil; R_f = 0.25 (*n*-hexane/EtOAc 1/1); ^{1}H NMR ($CDCl_3$, 400 MHz) δ 7.45 (d, *J* = 8.3 Hz, 2 ArH), 7.17–7.20 (m, 3H), 5.43–5.56 (m, trans–2H), 4.89 (s, NH), 3.71–3.75 (m, CH_2OH), 3.53–3.57 (m, CH_2OH), 3.35 (br, 2H), 2.88 (s, 2H), 2.41–2.42 (m, 4H), 1.96–2.01 (m, 2H), 1.44 (s, $C(CH_3)_3$), 1.25–1.28 (m, 22H), 0.87 (t, *J* = 7.0 Hz, CH_3).

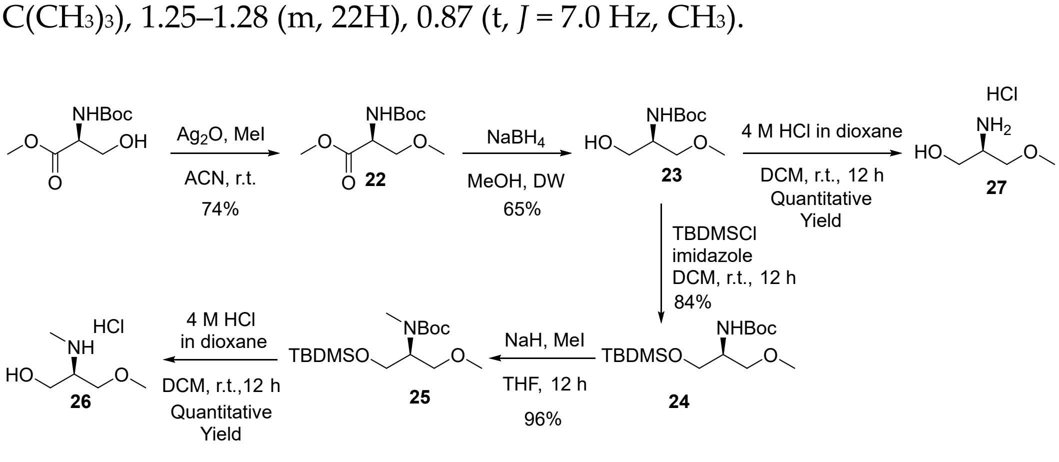

Scheme S1. Synthesis of **26** and **27**.

Synthesis of methyl *N*-(*tert*-butoxycarbonyl)-*O*-methyl-*L*-serinate (22) [2]

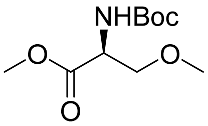

22

To a mixture of commercially available methyl (*tert*-butoxycarbonyl)-*L*-serinate (3.3 g, 15.0 mmol) in Acetonitrile (30 mL) was added Ag_2O (2.51 g, 36.5 mmol) then was added MeI. The resulting suspension was stirred at room temperature for 12 h. The mixture was filtered through a pad of Celite and the solvent was evaporated. The residue was purified by column chromatography to give **22** (2.6 g, 74%) as clear oil; R_f = 0.57 (*n*-hexane/EtOAc 1/1); ^{1}H NMR ($CDCl_3$, 400 MHz) δ 5.38–5.40 (m, 1H), 4.42–4.45 (m, 1H), 3.79–3.81 (m, 4H), 3.60–3.63 (m, 1H), 3.36 (s, OCH_3), 1.49 (s, $C(CH_3)_3$.

Synthesis of *tert*-butyl (*R*)-(1-hydroxy-3-methoxypropan-2-yl)carbamate (23) [2]

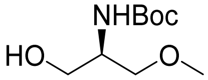

23

To a mixture of **22** (2.6 g, 11.1 mmol) in MeOH (11mL) and DW (11 mL) was slowly added sodium borohydride (843 mg, 22.2 mmol) at 0 °C. The resulting suspension was stirred at room temperature (12 h). The reaction mixture was diluted with distilled water and extracted with ethyl acetate. The combined organic layer was dried with anhydrous Na_2SO_4. The residue was purified by column chromatography to give **23** (1.5 g, 65%) as clear oil; R_f = 0.26 (*n*-hexane/EtOAc 1/1); ^{1}H NMR ($CDCl_3$, 400 MHz) δ 5.17 (br, 1H), 3.75–3.77 (m, 2H), 3.65–3.67 (m, 1H), 3.50–3.56 (m, 2H), 3.34 (s, OCH_3), 2.81–2.84 (m, 1H), 1.45 (s, $(CH_3)_3$); ^{13}C NMR ($CDCl_3$, 100 MHz) δ 156.0, 79.6, 73.2, 64.0, 59.2, 51.4, 28.3.

Synthesis of *tert*-butyl (*S*)-(1-((tert-butyldimethylsilyl)oxy)-3-methoxypropan-2-yl)carbamate (24) [2]

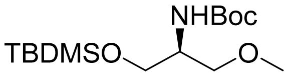

24

To a mixture of **23** (1.3 g, 6.33 mmol) in dichloro methane (20 mL) was added TBDMSCl (1.9 g, 12.9 mmol) then was added imidazole (904 mg, 13.29 mmol) portionwise at 0 °C (5 min). The resulting suspension was stirred at room temperature (12 h). The reaction mixture was diluted with distilled water and extracted with ethyl acetate. The combined organic layer was dried with anhydrous Na_2SO_4. The residue was purified by column chromatography to give **24** (1.7 g, 84%) as clear oil; R_f = 0.73 (*n*-hexane/EtOAc 1/1); $[\alpha]_D^{25}$ = -9.20 (*c* = 0.51, $CHCl_3$); ^{1}H NMR ($CDCl_3$, 400 MHz) δ 4.83–4.84 (m, 1H), 3.68–3.74 (m, 2H), 3.54–3.58 (m, 1H), 3.43–3.47 (m, 1H), 3.33–3.37 (m, 1H), 3.32 (s, OCH_3), 1.42 (s, 9H), 0.87 (s, 9H), 0.04 (s, 6H); ^{13}C NMR ($CDCl_3$, 100 MHz) δ 155.4, 79.2, 70.6, 61.5, 58.8, 51.0, 28.3, 25.8, 25.6, 18.2, 5.4, 5.5.

Synthesis of tert-butyl (*S*)-(1-((tert-butyldimethylsilyl)oxy)-3-methoxypropan-2-yl)(methyl)carbamate (25) [2]

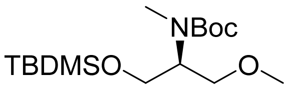

25

To a mixture of **24** (1.7 g, 5.6 mmol) in anhydrous tetrahydrofuran (17 mL) was added 60% sodium hydride (636 mg, 15.68 mmol) at 0 °C then was added iodomethane (0.22 mL, 13.29 mmol) and DMF (0.2 mL) at 0 °C (5 min). The resulting suspension was stirred at room temperature (12 h). The reaction mixture was diluted with distilled water and extracted with ethyl acetate. The combined organic layer was dried with anhydrous Na_2SO_4. The residue was purified by column chromatography to give **25** (1.8 g, 96%) as clear oil; R_f = 0.68 (*n*-hexane/EtOAc 1/1); $[\alpha]_D^{25}$ = -5.57 (*c* = 0.70, $CHCl_3$); ^1H NMR ($CDCl_3$, 400 MHz) δ 4.14–4.25 (m, 1H), 3.66–3.72 (m, 2H), 3.49–3.57 (m, 2H), 3.32 (s, OCH_3), 2.83(s, NCH_3), 1.45 (s, 9H), 0.87 (s, 9H), 0.04 (s, 6H).

Synthesis of (*R*)-3-methoxy-2-(methylamino)propan-1-ol hydrogen chloride (26)

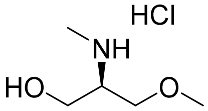

26

Using Method B, **25** (5.85 g, 17.5 mmol) and 4 M HCl in dioxane (22 mL, 87.6 mmol) gave 3.2 g (quantitative yield) of **26** as yellow oil; $[\alpha]_D^{25}$ = -3.56 (*c* = 1.32, EtOH); ^1H NMR ($CDCl_3$, 400 MHz) δ 8.98 (br, NHHCl), 4.88 (br, OH), 3.76–3.98 (m, 4H), 3.64–3.38 (m, 4H), 2.82 (s, $NHCH_3$); ^{13}C NMR ($CDCl_3$, 100 MHz) δ 69.1, 61.1, 59.2, 59.0, 32.1.

Synthesis of (*R*)-2-amino-3-methoxypropan-1-ol hydrochloride (27)

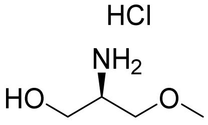

27

Using Method B, **23** (224 mg, 1.09 mmol) and 4 M HCl in dioxane (0.81 mL, 3.27 mmol) gave 200 mg (quantitative yield) of **27** as yellow oil; ^1H NMR (CD_3OD, 400 MHz) δ 3.56–3.77 (m, 4H), 3.31 (s, OCH_3), 3.30–3.31 (m, 1H); ^{13}C NMR (CD_3OD, 100 MHz) δ 69.1, 58.7, 58.1, 52.7.

3. ^{1}H NMR and ^{13}C NMR spectra for the final compounds

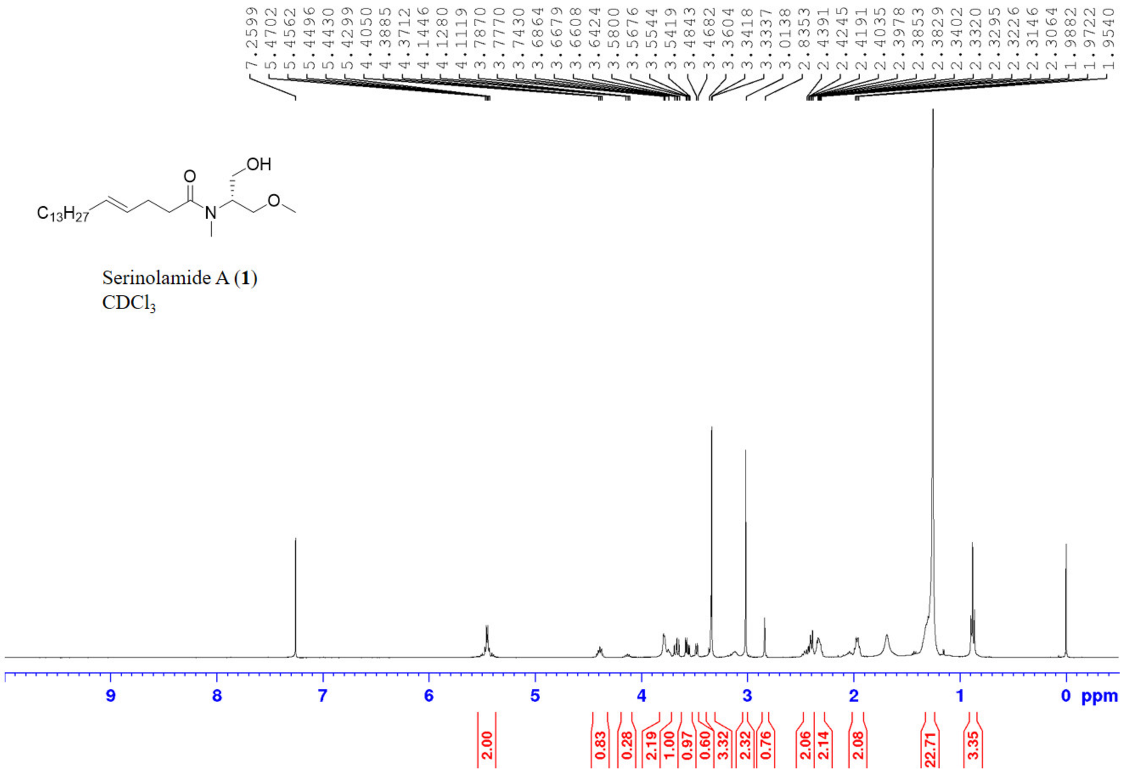

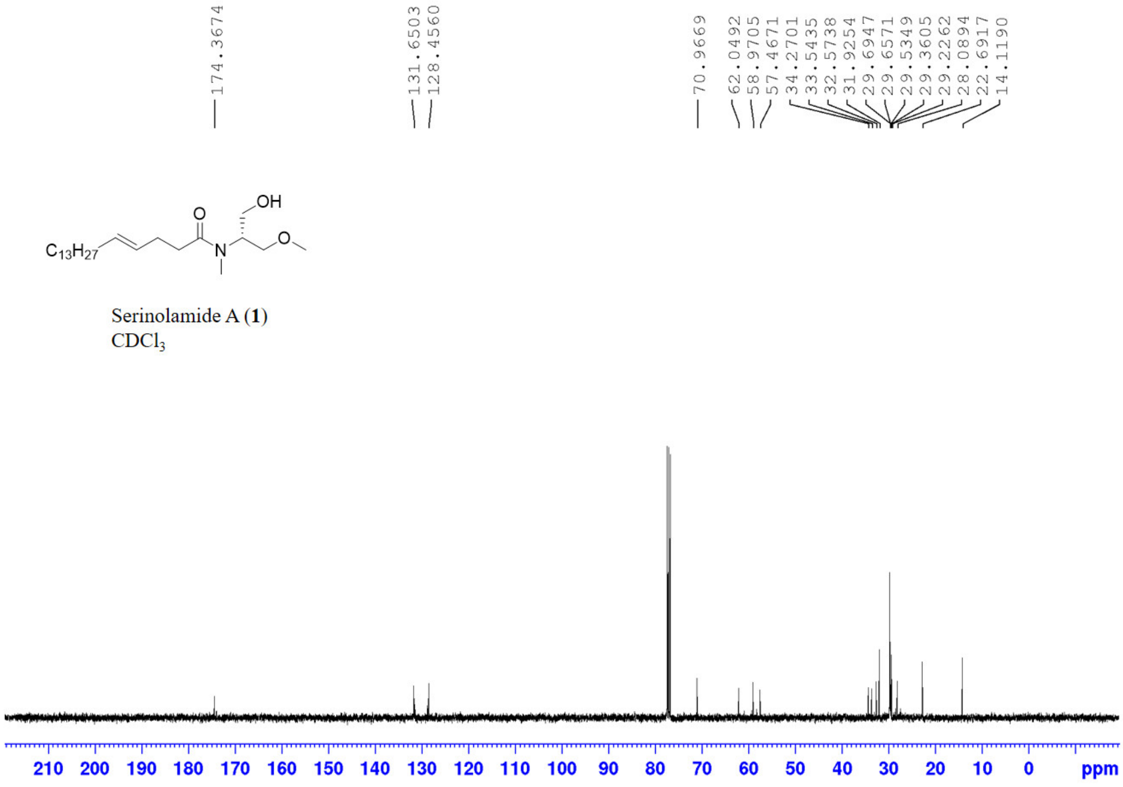

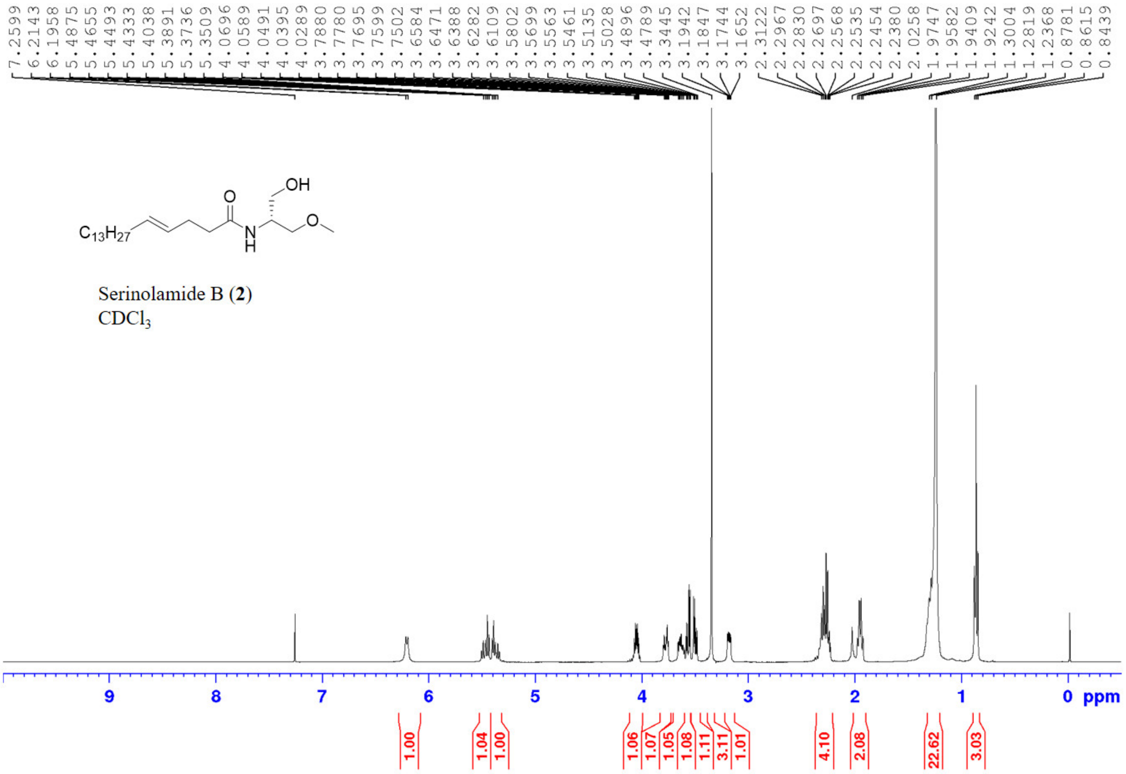
7.2599
6.2143
6.1958
5.4875
5.4655
5.4493
5.4333
5.4038
5.3891
5.3736
5.3509
4.0696
4.0589
4.0491
4.0395
4.0289
3.7880
3.7780
3.7695
3.7599
3.7502
3.6584
3.6471
3.6388
3.6282
3.6109
3.5802
3.5699
3.5563
3.5461
3.5135
3.5028
3.4896
3.4789
3.3445
3.1942
3.1847
3.1744
3.1652
2.3122
2.2967
2.2830
2.2697
2.2568
2.2535
2.2454
2.2380
2.0258
1.9747
1.9582
1.9409
1.9242
1.3004
1.2819
1.2368
0.8781
0.8615
0.8439
OH
O
$C_{13}H_{27}$
N
H
O
Serinolamide B (**2**)
$CDCl_3$
9
8
7
6
5
4
3
2
1
0 ppm
1.00
1.04
1.00
1.06
1.07
1.05
1.08
1.11
3.11
1.01
4.10
2.08
22.62
3.03

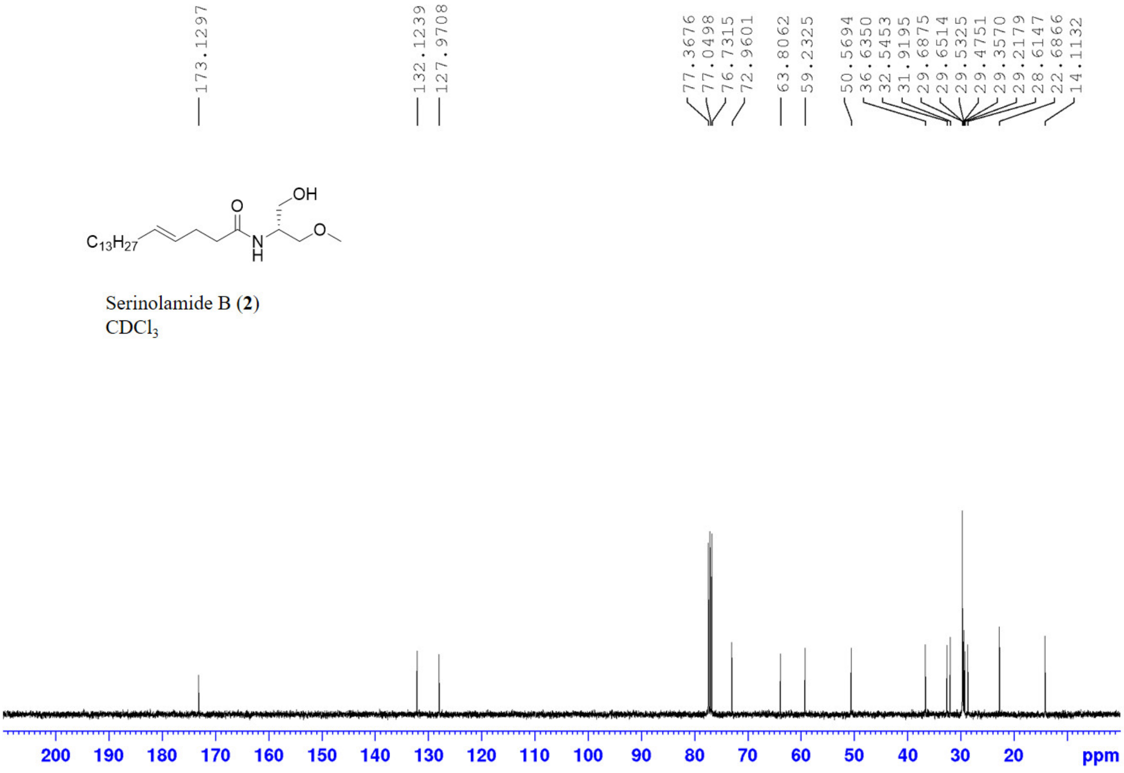
173.1297
132.1239
127.9708
77.3676
77.0498
76.7315
72.9601
63.8062
59.2325
50.5694
36.6350
32.5453
31.9195
29.6875
29.6514
29.5325
29.4751
29.3570
29.2179
28.6147
22.6866
14.1132
OH
O
$C_{13}H_{27}$
N
H
O
Serinolamide B (**2**)
$CDCl_3$
200
190
180
170
160
150
140
130
120
110
100
90
80
70
60
50
40
30
20
ppm

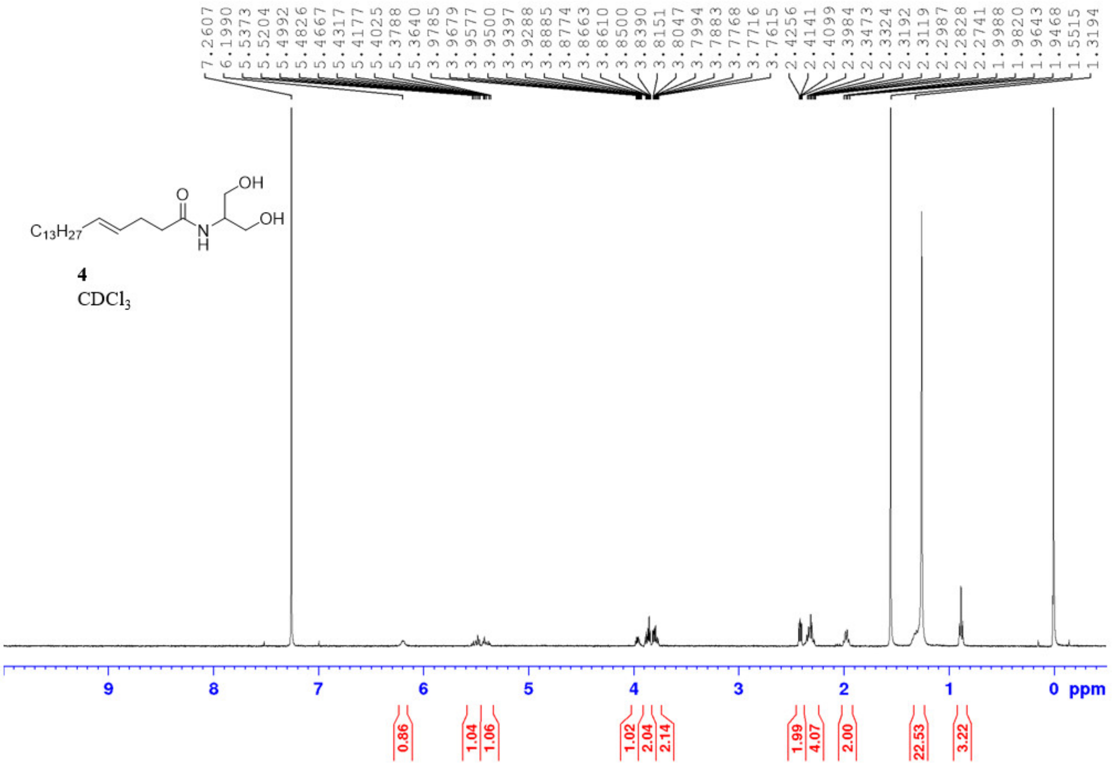

7.2607
6.1990
5.5373
5.5204
5.4992
5.4826
5.4667
5.4317
5.4177
5.4025
5.3788
5.3640
3.9785
3.9679
3.9577
3.9500
3.9397
3.9288
3.8885
3.8774
3.8663
3.8610
3.8500
3.8390
3.8151
3.8047
3.7994
3.7883
3.7768
3.7716
3.7615
2.4256
2.4141
2.4099
2.3984
2.3473
2.3324
2.3192
2.3119
2.2987
2.2828
2.2741
1.9988
1.9820
1.9643
1.9468
1.5515
1.3194
$C_{13}H_{27}$
O
N
H
OH
OH
4
$CDCl_3$
9 8 7 6 5 4 3 2 1 0 ppm
0.86
1.04
1.06
1.02
2.04
2.14
1.99
4.07
2.00
22.53
3.22

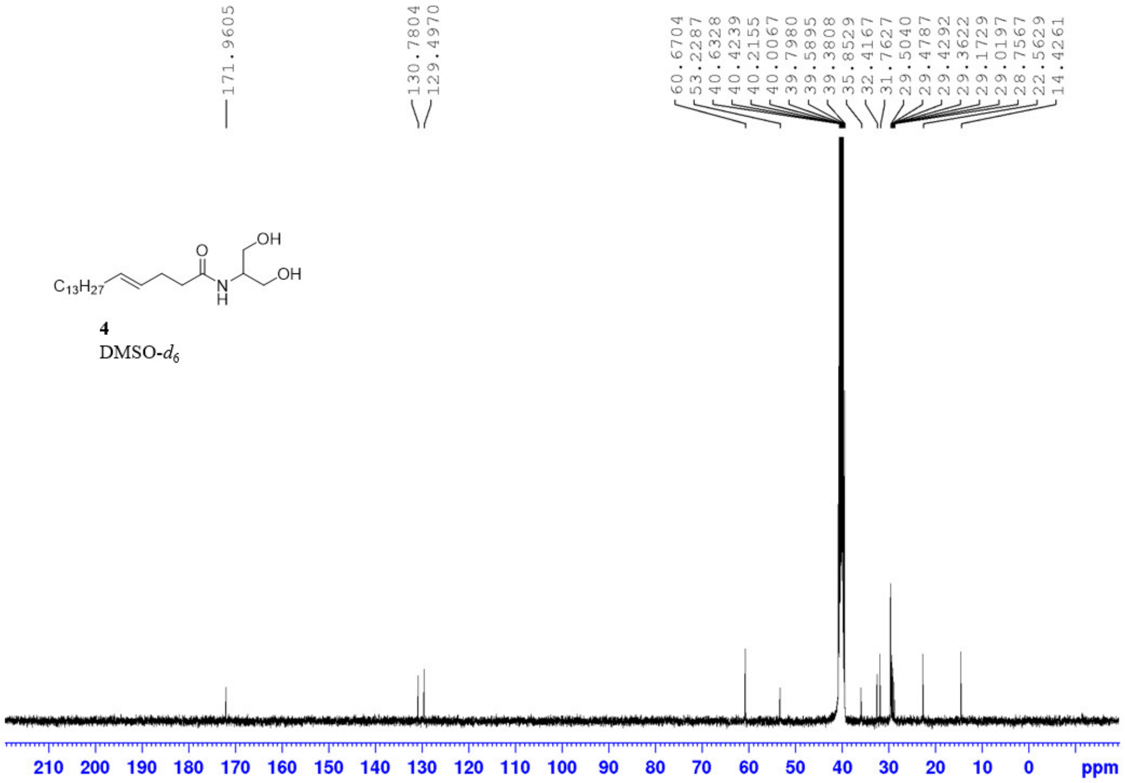

171.9605
130.7804
129.4970
60.6704
53.2287
40.6328
40.4239
40.2155
40.0067
39.7980
39.5895
39.3808
35.8529
32.4167
31.7627
29.5040
29.4787
29.4292
29.3622
29.1729
29.0197
28.7567
22.5629
14.4261
$C_{13}H_{27}$
O
N
H
OH
OH
4
DMSO-d_6
210 200 190 180 170 160 150 140 130 120 110 100 90 80 70 60 50 40 30 20 10 0 ppm

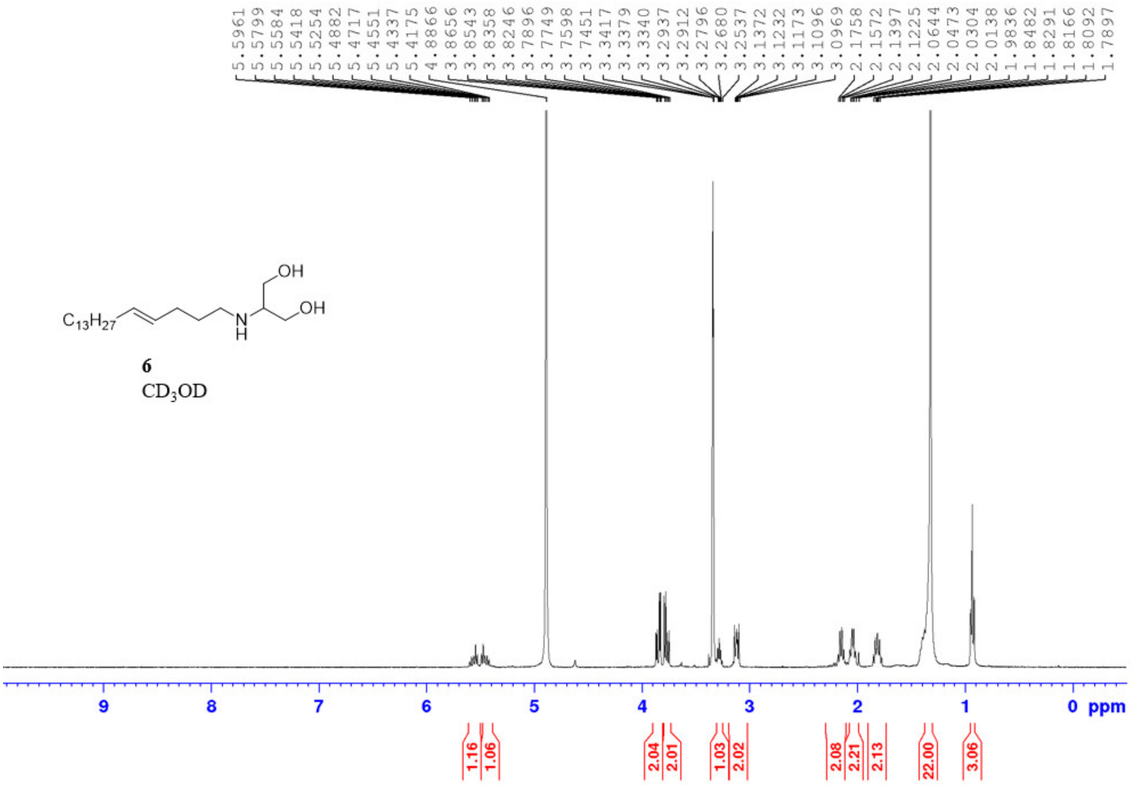
5.5961
5.5799
5.5584
5.5418
5.5254
5.4882
5.4717
5.4551
5.4337
5.4175
4.8866
3.8656
3.8543
3.8358
3.8246
3.7896
3.7749
3.7598
3.7451
3.3417
3.3379
3.3340
3.2937
3.2912
3.2796
3.2680
3.2537
3.1372
3.1232
3.1173
3.1096
3.0969
2.1758
2.1572
2.1397
2.1225
2.0644
2.0473
2.0304
2.0138
1.9836
1.8482
1.8291
1.8166
1.8092
1.7897
$C_{13}H_{27}$
N
H
OH
OH
6
CD_3OD
1.16
1.06
2.04
2.01
1.03
2.02
2.08
2.21
2.13
22.00
3.06
ppm

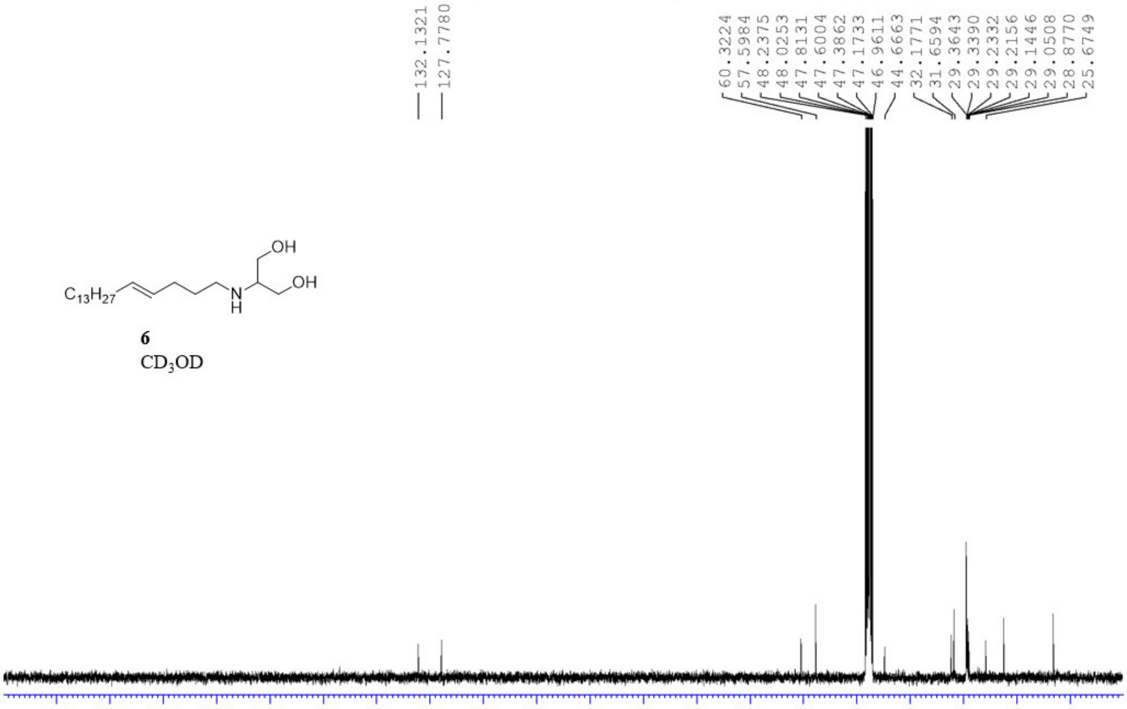
132.1321
127.7780
60.3224
57.5984
48.2375
48.0253
47.8131
47.6004
47.3862
47.1733
46.9611
44.6663
32.1771
31.6594
29.3643
29.3390
29.2332
29.2156
29.1446
29.0508
28.8770
25.6749
$C_{13}H_{27}$
N
H
OH
OH
6
CD_3OD
ppm

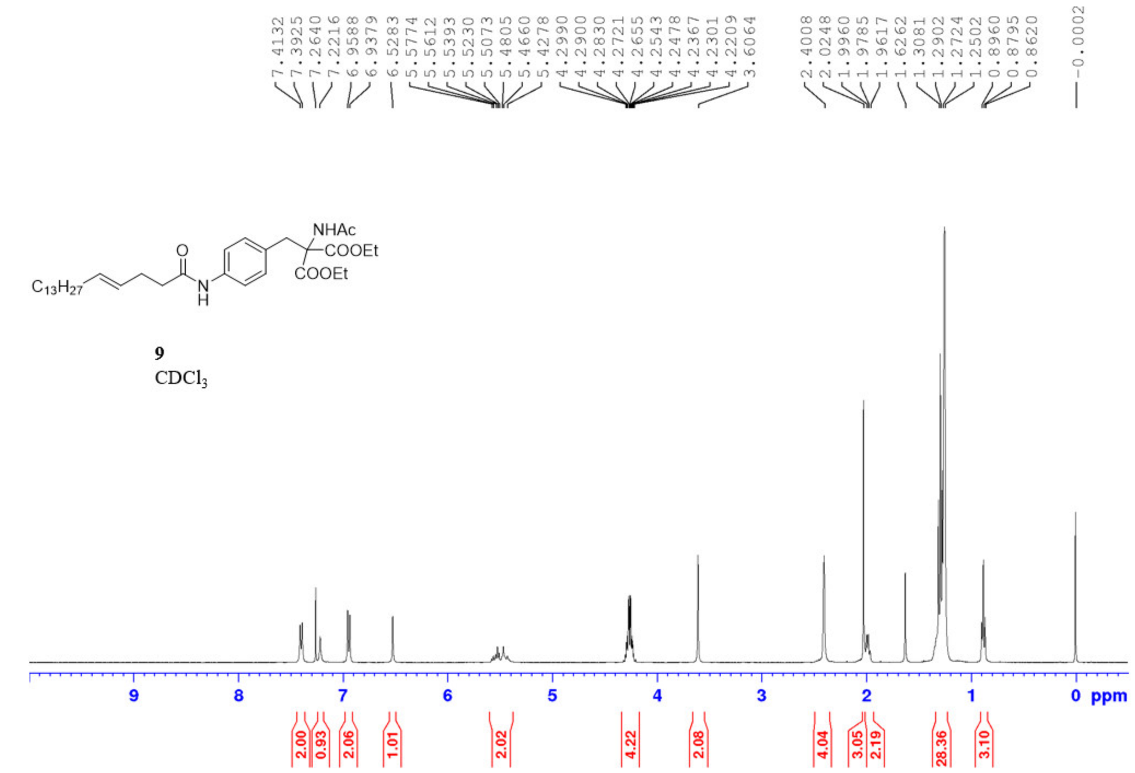

7.4132
7.3925
7.2640
7.2216
6.9588
6.9379
6.5283
5.5774
5.5612
5.5393
5.5230
5.5073
5.4805
5.4660
5.4278
4.2990
4.2900
4.2830
4.2721
4.2655
4.2543
4.2478
4.2367
4.2301
4.2209
3.6064
2.4008
2.0248
1.9960
1.9785
1.9617
1.6262
1.3081
1.2902
1.2724
1.2502
0.8960
0.8795
0.8620
-0.0002
NHAc
COOEt
COOEt
O
N
H
$C_{13}H_{27}$
9
$CDCl_3$
9
8
7
6
5
4
3
2
1
0 ppm
2.00
0.93
2.06
1.01
2.02
4.22
2.08
4.04
3.05
2.19
28.36
3.10

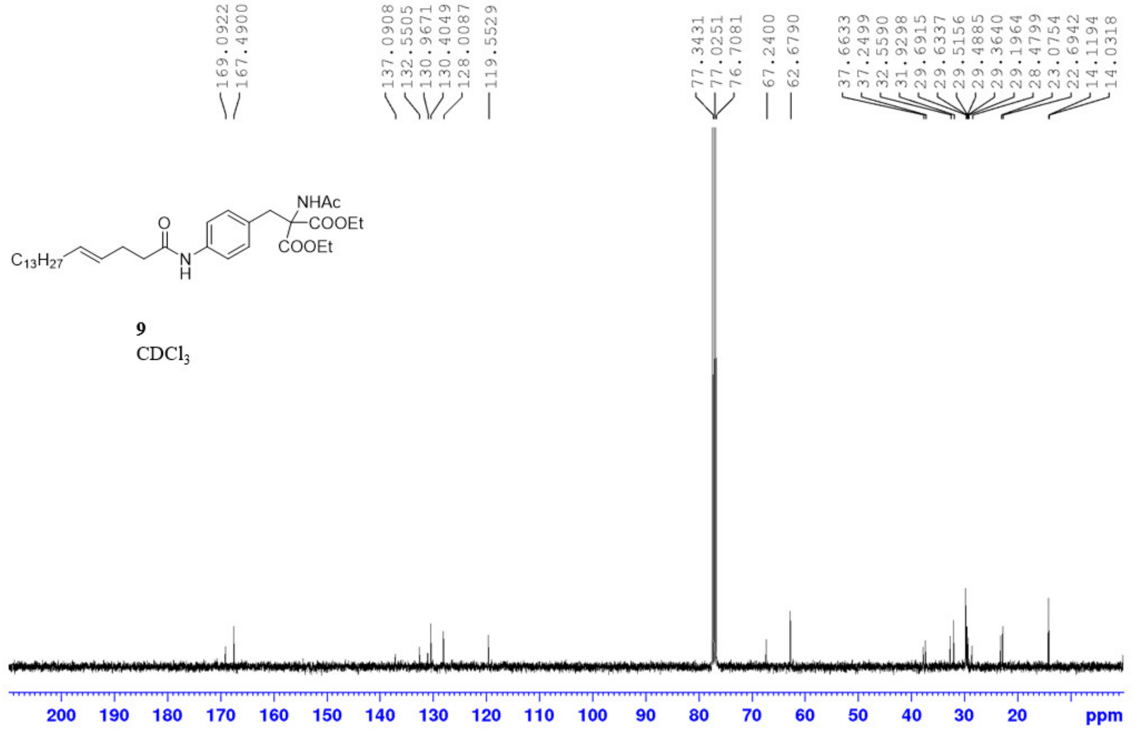

169.0922
167.4900
137.0908
132.5505
130.9671
130.4049
128.0087
119.5529
77.3431
77.0251
76.7081
67.2400
62.6790
37.6633
37.2499
32.5590
31.9298
29.6915
29.6337
29.5156
29.4885
29.3640
29.1964
28.4799
23.0754
22.6942
14.1194
14.0318
NHAc
COOEt
COOEt
O
N
H
$C_{13}H_{27}$
9
$CDCl_3$
200
190
180
170
160
150
140
130
120
110
100
90
80
70
60
50
40
30
20
ppm

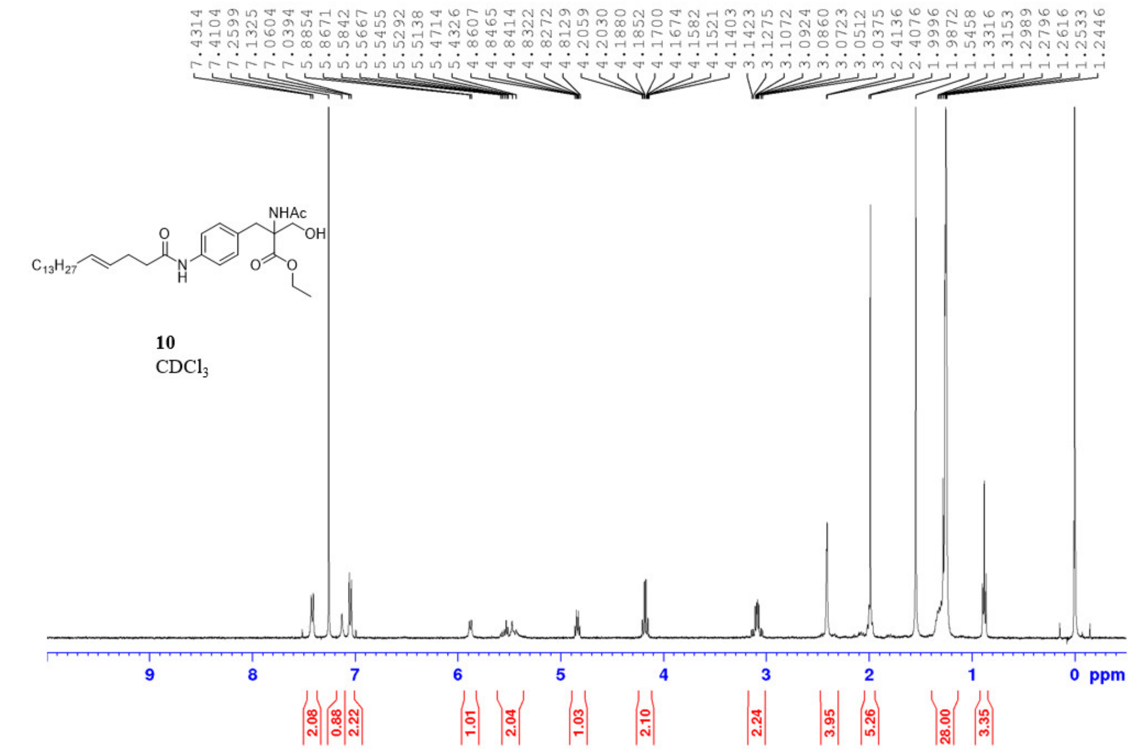
7.4314 7.4104 7.2599 7.1325 7.0604 7.0394 5.8854 5.8671 5.5842 5.5667 5.5455 5.5292 5.5138 5.4714 5.4326 4.8607 4.8465 4.8414 4.8322 4.8272 4.8129 4.2059 4.2030 4.1880 4.1852 4.1700 4.1674 4.1582 4.1521 4.1403 3.1423 3.1275 3.1072 3.0924 3.0860 3.0723 3.0512 3.0375 2.4136 2.4076 1.9996 1.9872 1.5458 1.3316 1.3153 1.2989 1.2796 1.2616 1.2533 1.2446
NHAc
OH
$C_{13}H_{27}$
N
H
O
O
O
10
$CDCl_3$
9 8 7 6 5 4 3 2 1 0 ppm
2.08 0.88 2.22 1.01 2.04 1.03 2.10 2.24 3.95 5.26 28.00 3.35

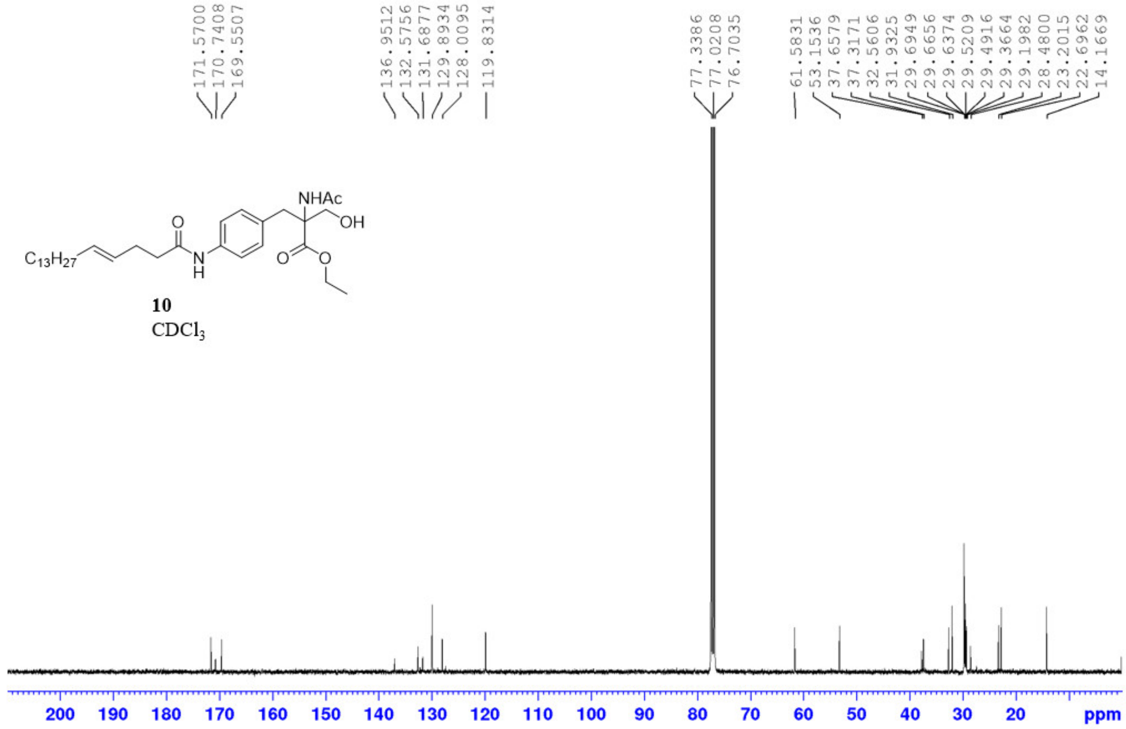
171.5700 170.7408 169.5507 136.9512 132.5756 131.6877 129.8934 128.0095 119.8314 77.3386 77.0208 76.7035 61.5831 53.1536 37.6579 37.3171 32.5606 31.9325 29.6949 29.6656 29.6374 29.5209 29.4916 29.3664 29.1982 28.4800 23.2015 22.6962 14.1669
NHAc
OH
$C_{13}H_{27}$
N
H
O
O
O
10
$CDCl_3$
200 190 180 170 160 150 140 130 120 110 100 90 80 70 60 50 40 30 20 ppm

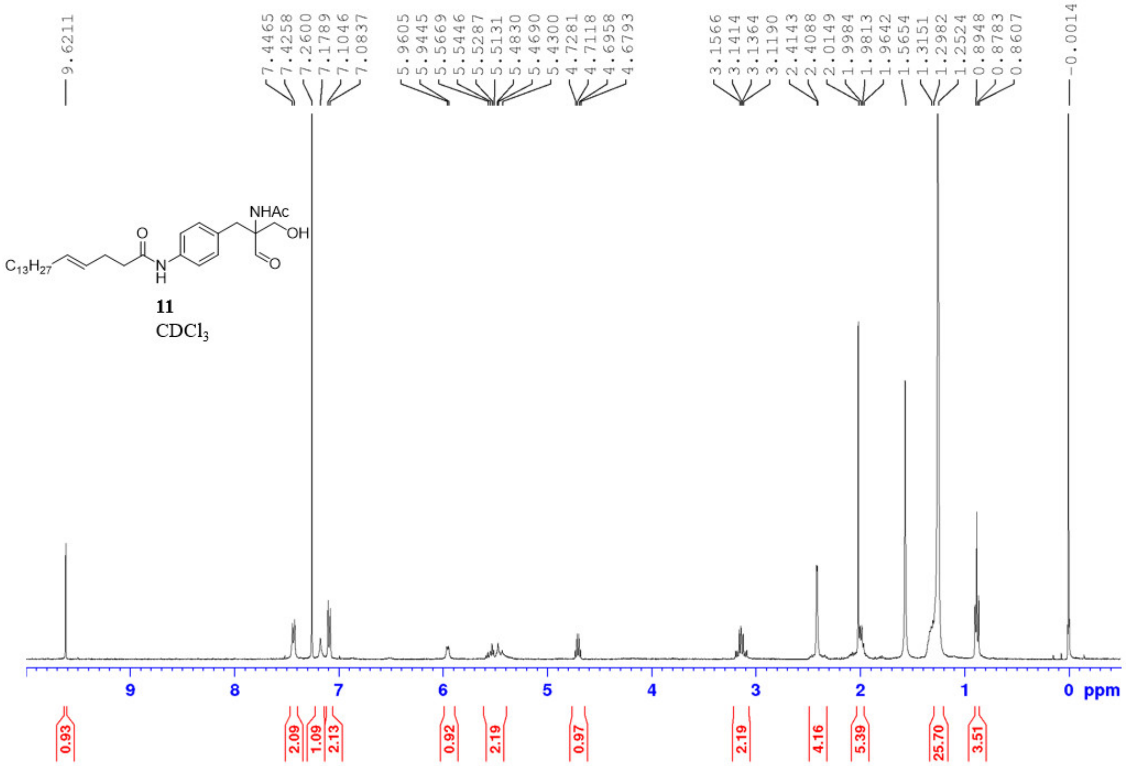

9.6211
7.4465
7.4258
7.2600
7.1789
7.1046
7.0837
5.9605
5.9445
5.5669
5.5446
5.5287
5.5131
5.4830
5.4690
5.4300
4.7281
4.7118
4.6958
4.6793
3.1566
3.1414
3.1364
3.1190
2.4143
2.4088
2.0149
1.9984
1.9813
1.9642
1.5654
1.3151
1.2982
1.2524
0.8948
0.8783
0.8607
-0.0014
NHAc
OH
O
$C_{13}H_{27}$
N
H
O
11
$CDCl_3$
9 8 7 6 5 4 3 2 1 0 ppm
0.93
2.09
1.09
2.13
0.92
2.19
0.97
2.19
4.16
5.39
25.70
3.51

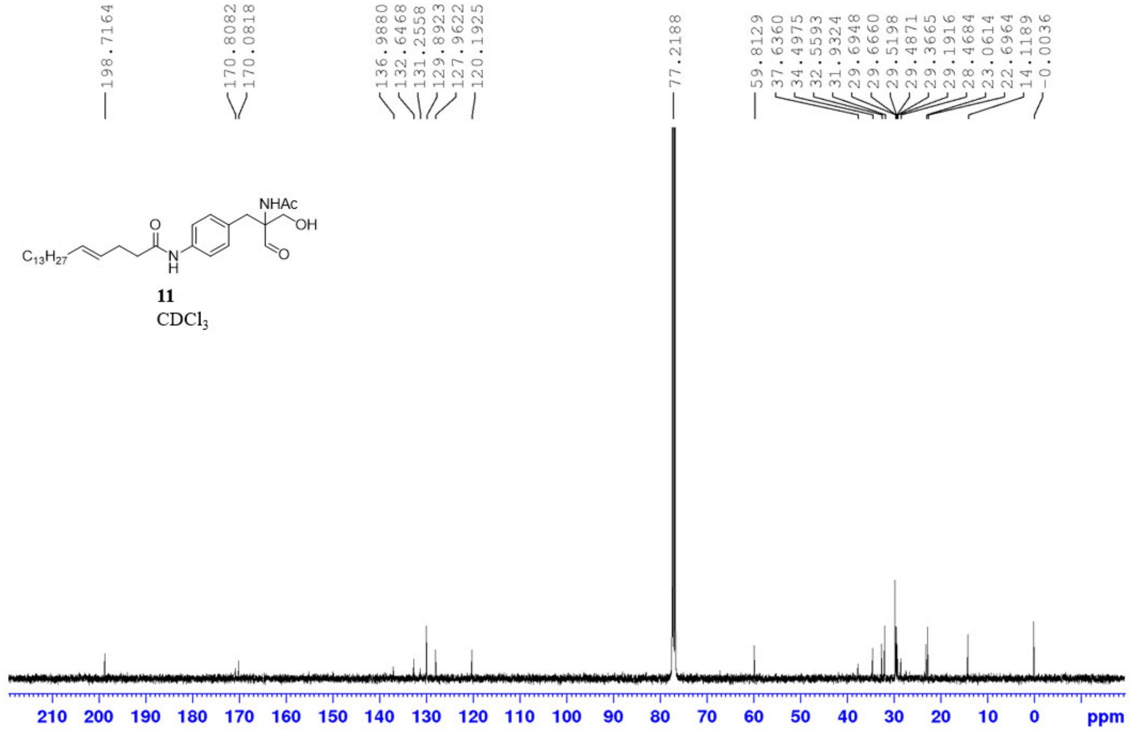

198.7164
170.8082
170.0818
136.9880
132.6468
131.2558
129.8923
127.9622
120.1925
77.2188
59.8129
37.6360
34.4975
32.5593
31.9324
29.6948
29.6660
29.5198
29.4871
29.3665
29.1916
28.4684
23.0614
22.6964
14.1189
-0.0036
NHAc
OH
O
$C_{13}H_{27}$
N
H
O
11
$CDCl_3$
210 200 190 180 170 160 150 140 130 120 110 100 90 80 70 60 50 40 30 20 10 0 ppm

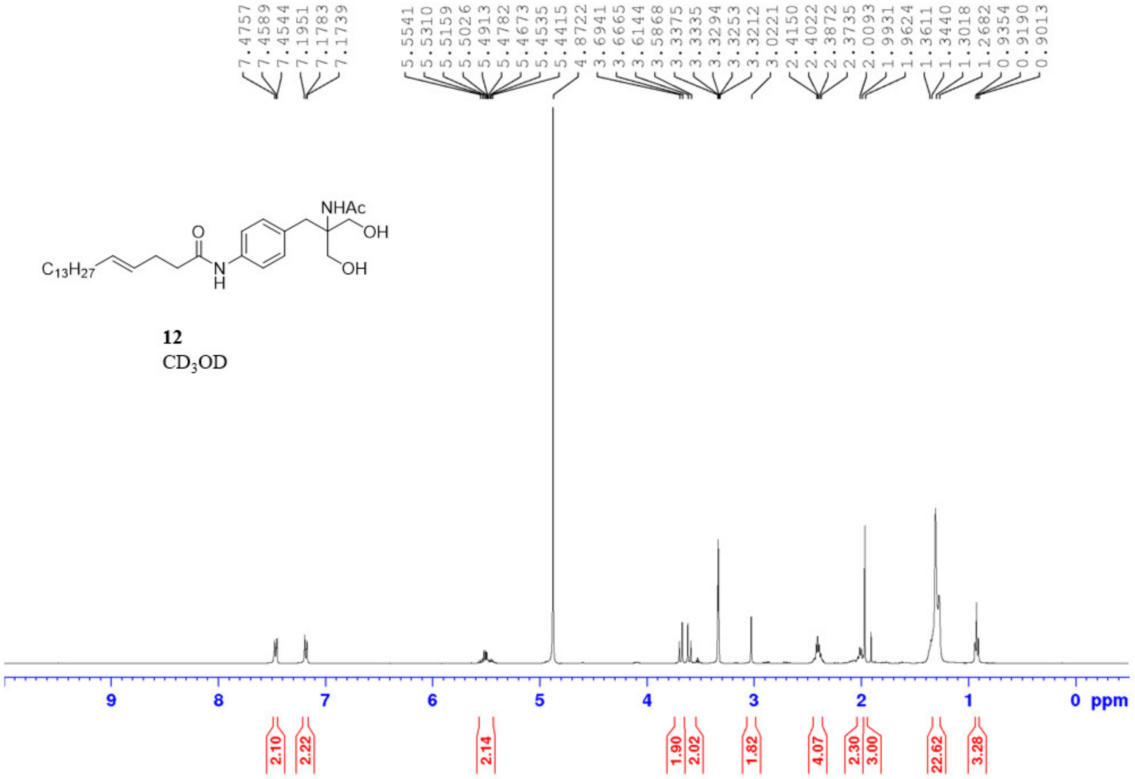

12
CD_3OD

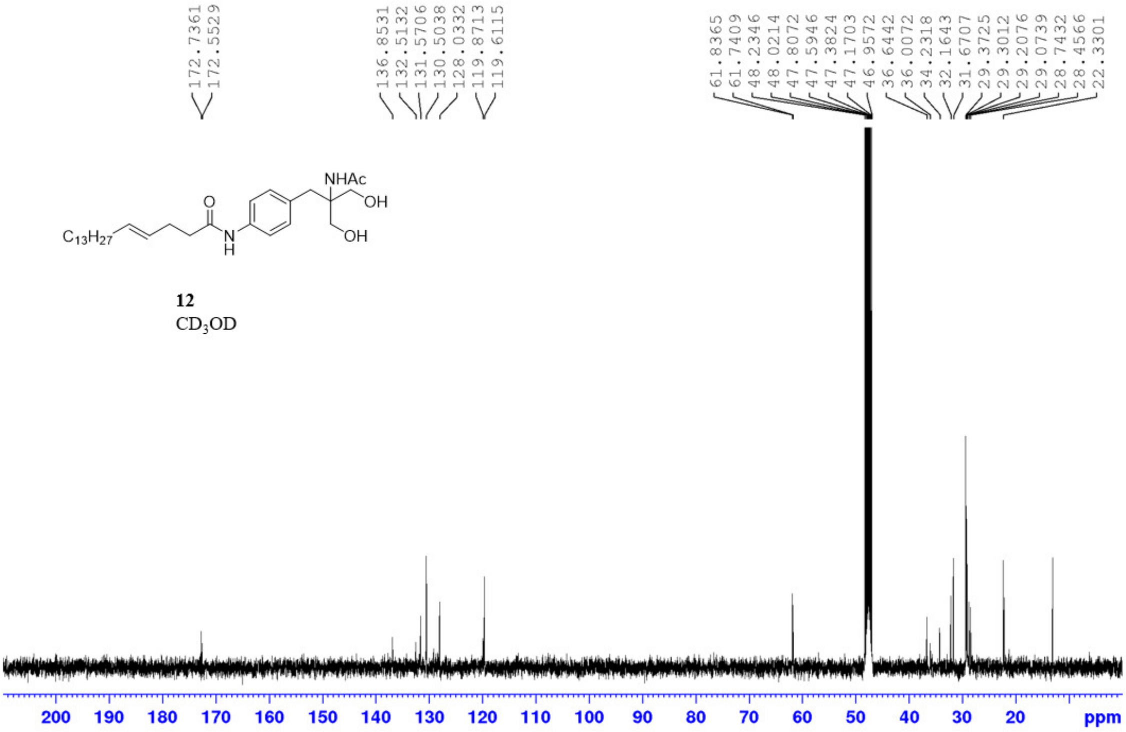

12
CD_3OD

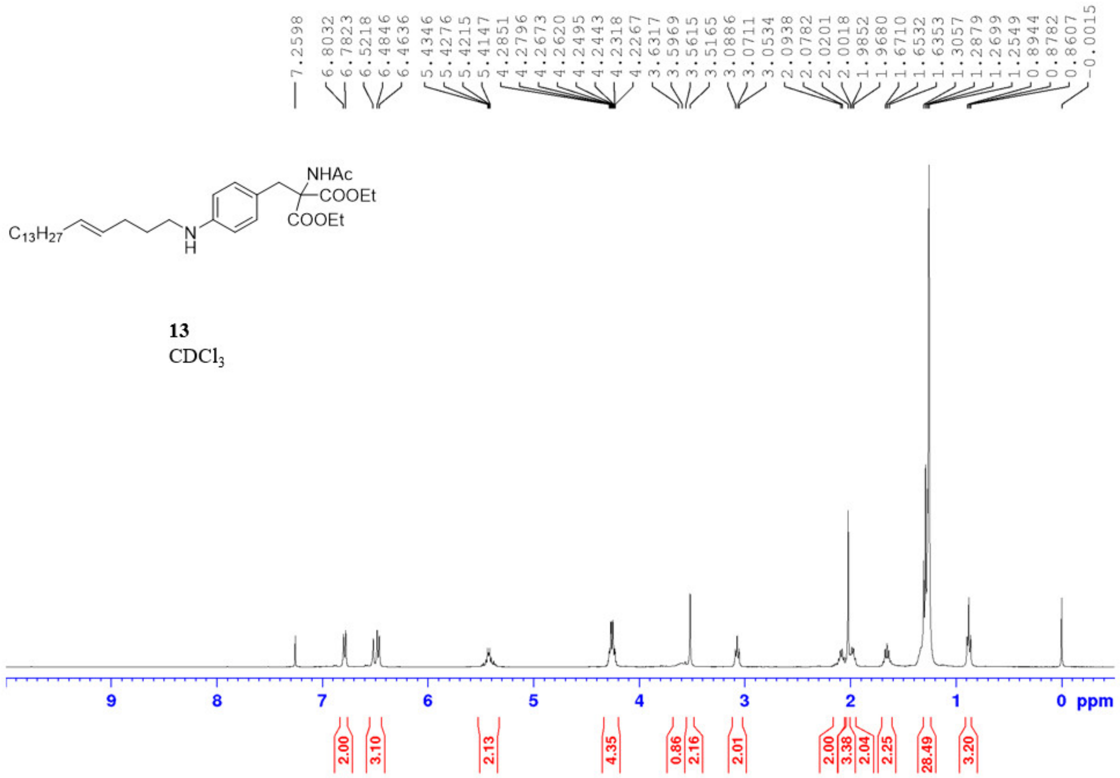

13
$CDCl_3$

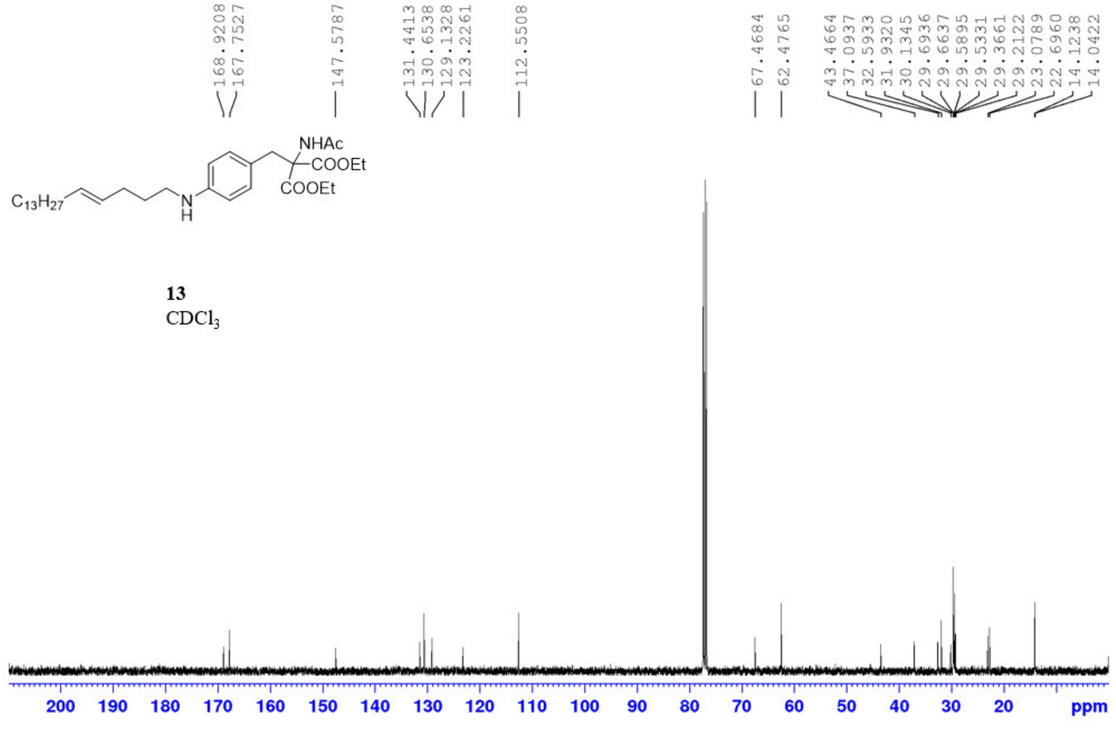

13
$CDCl_3$

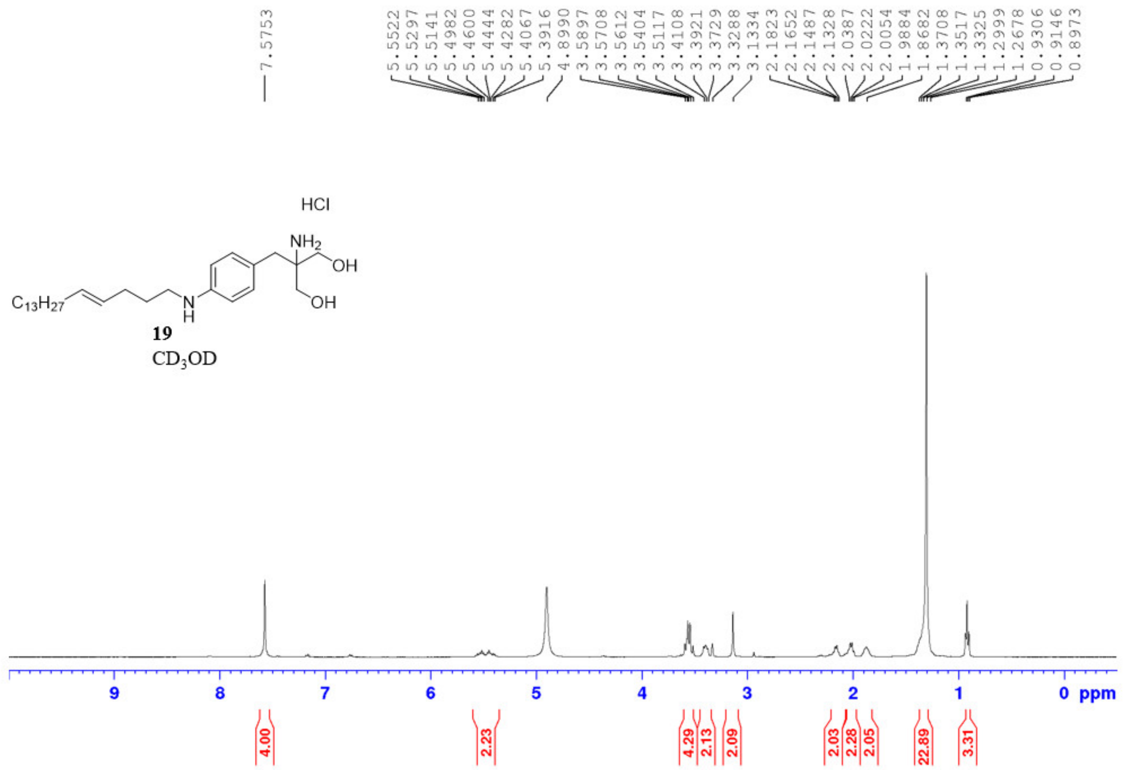
7.5753
5.5522
5.5297
5.5141
5.4982
5.4600
5.4444
5.4282
5.4067
5.3916
4.8990
3.5897
3.5708
3.5612
3.5404
3.5117
3.4108
3.3921
3.3729
3.3288
3.1334
2.1823
2.1652
2.1487
2.1328
2.0387
2.0222
2.0054
1.9884
1.8682
1.3708
1.3517
1.3325
1.2999
1.2678
0.9306
0.9146
0.8973
HCl
NH2
OH
OH
C13H27
N
H
19
CD3OD
9 8 7 6 5 4 3 2 1 0 ppm
4.00
2.23
4.29
2.13
2.09
2.03
2.28
2.05
22.89
3.31

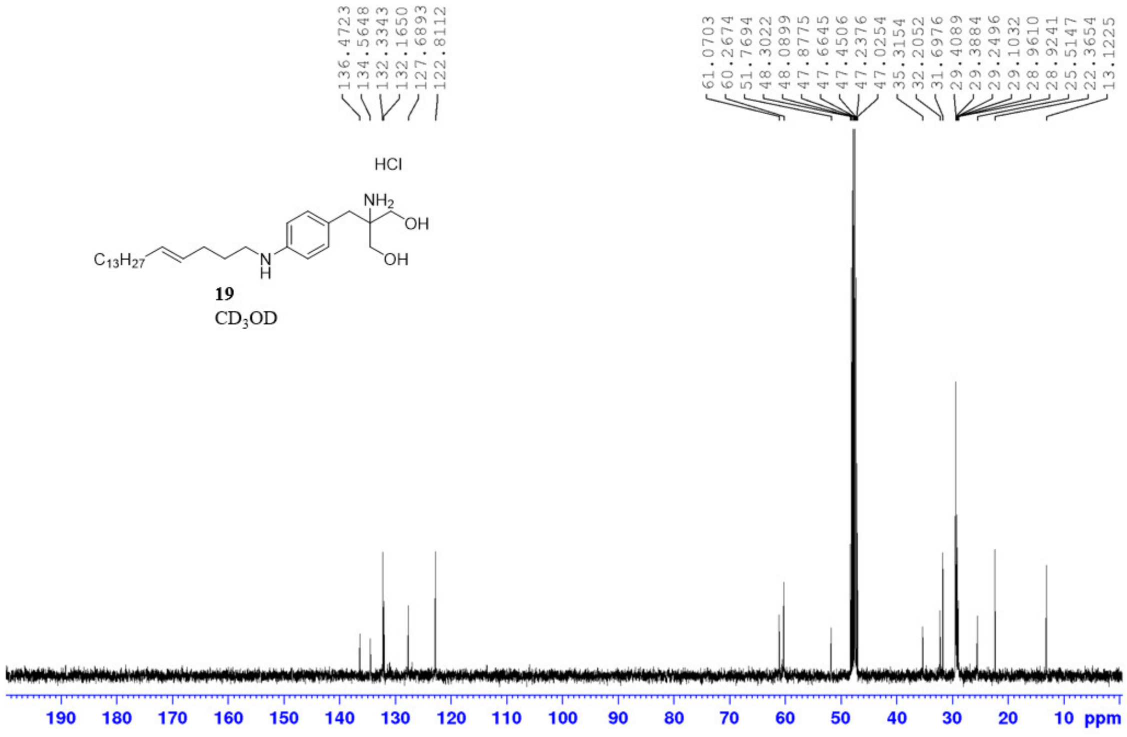
136.4723
134.5648
132.3343
132.1650
127.6893
122.8112
61.0703
60.2674
51.7694
48.3022
48.0899
47.8775
47.6645
47.4506
47.2376
47.0254
35.3154
32.2052
31.6976
29.4089
29.3884
29.2496
29.1032
28.9610
28.9241
25.5147
22.3654
13.1225
HCl
NH2
OH
OH
C13H27
N
H
19
CD3OD
190 180 170 160 150 140 130 120 110 100 90 80 70 60 50 40 30 20 10 ppm

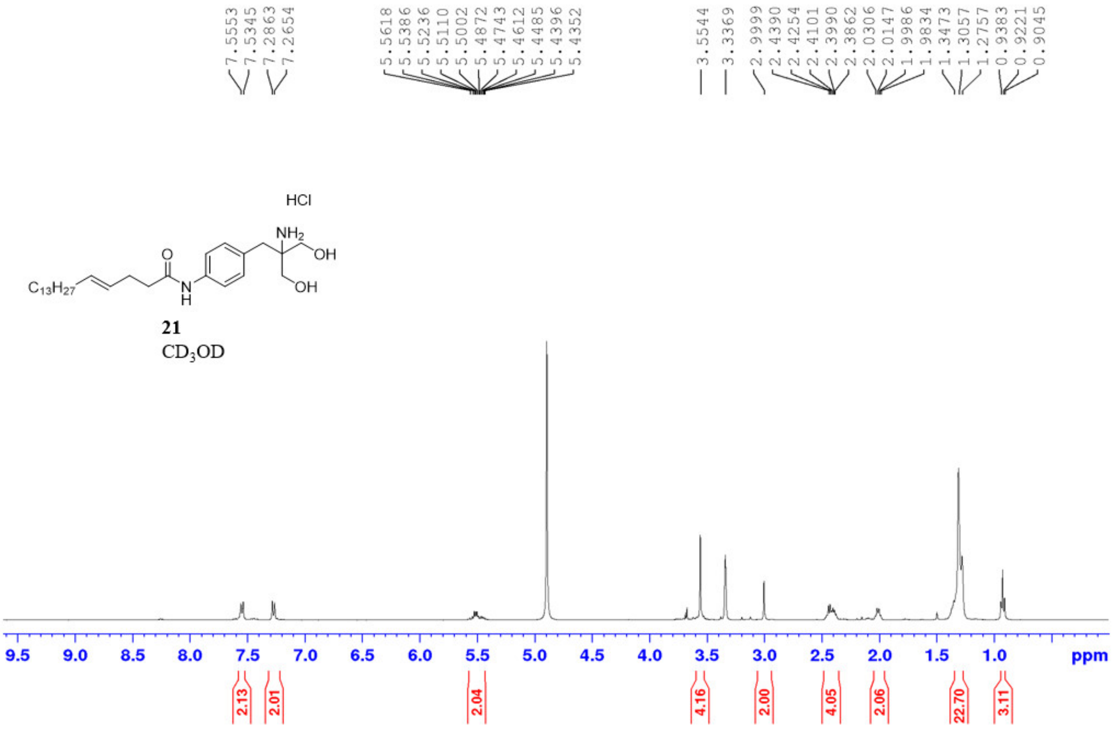
7.5553
7.5345
7.2863
7.2654
5.5618
5.5386
5.5236
5.5110
5.5002
5.4872
5.4743
5.4612
5.4485
5.4396
5.4352
3.5544
3.3369
2.9999
2.4390
2.4254
2.4101
2.3990
2.3862
2.0306
2.0147
1.9986
1.9834
1.3473
1.3057
1.2757
0.9383
0.9221
0.9045
HCl
NH2
OH
OH
O
N
H
C13H27
21
CD3OD
9.5 9.0 8.5 8.0 7.5 7.0 6.5 6.0 5.5 5.0 4.5 4.0 3.5 3.0 2.5 2.0 1.5 1.0 ppm
2.13
2.01
2.04
4.16
2.00
4.05
2.06
22.70
3.11

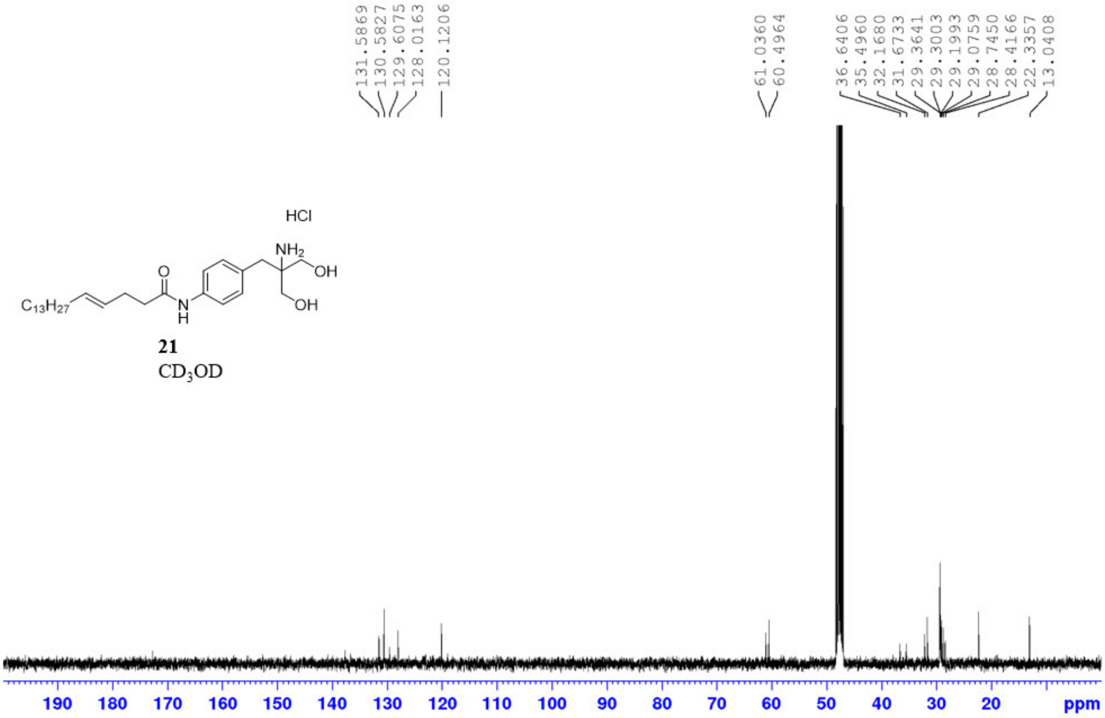
131.5869
130.5827
129.6075
128.0163
120.1206
61.0360
60.4964
36.6406
35.4960
32.1680
31.6733
29.3641
29.3003
29.1993
29.0759
28.7450
28.4166
22.3357
13.0408
HCl
NH2
OH
OH
O
N
H
C13H27
21
CD3OD
190 180 170 160 150 140 130 120 110 100 90 80 70 60 50 40 30 20 ppm

HPLC

SAMPLE INFORMATION

Sample Name:	8126	Acquired By:	System
Sample Type:	Unknown	Sample Set Name	General method set_10
Vial:	8	Acq. Method Set:	General_A
Injection #:	1	Processing Methoc	General_A
Injection Volume:	20.00 ul	Channel Name:	W2489 ChA
Run Time:	20.0 Minutes	Proc. Chnl. Descr.:	W2489 ChA 254nm
Date Acquired:	12/9/2021 12:07:41 PM KST		
Date Processed:	12/9/2021 12:41:02 PM KST		

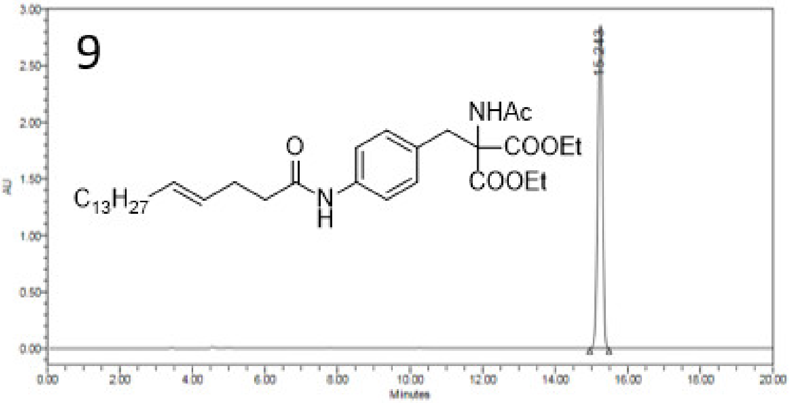

	RT	Area	% Area	Height
1	15.243	25711154	100.00	2871887

SAMPLE INFORMATION

Sample Name:	8127	Acquired By:	System
Sample Type:	Unknown	Sample Set Name	General method set_10
Vial:	12	Acq. Method Set:	General_A
Injection #:	1	Processing Methoc	General_A
Injection Volume:	20.00 ul	Channel Name:	W2489 ChA
Run Time:	20.0 Minutes	Proc. Chnl. Descr.:	W2489 ChA 254nm
Date Acquired:	12/8/2021 8:14:44 PM KST		
Date Processed:	12/9/2021 8:48:56 AM KST		

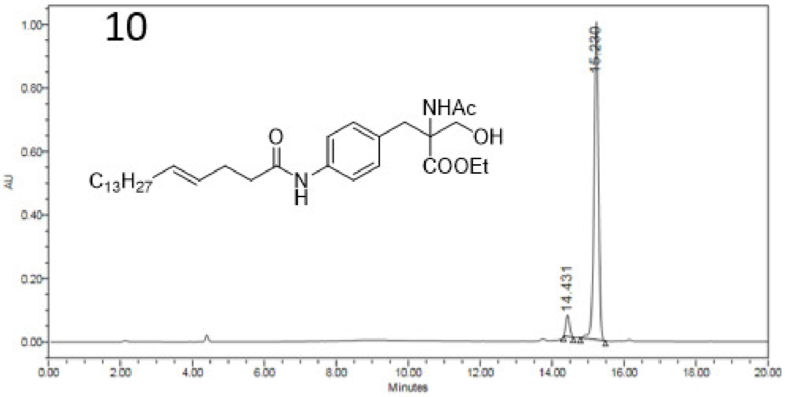

	RT	Area	% Area	Height
1	14.431	497139	5.43	67550
2	15.230	8663503	94.57	1000107

SAMPLE INFORMATION

Sample Name:	8128	Acquired By:	System
Sample Type:	Unknown	Sample Set Name	a
Vial:	51	Acq. Method Set:	General_A
Injection #:	1	Processing Method	General_A
Injection Volume:	30.00 ul	Channel Name:	W2489 ChA
Run Time:	20.0 Minutes	Proc. Chnl. Descr.:	W2489 ChA 254nm

Date Acquired: 12/10/2021 2:01:36 PM KST
Date Processed: 12/10/2021 2:55:11 PM KST

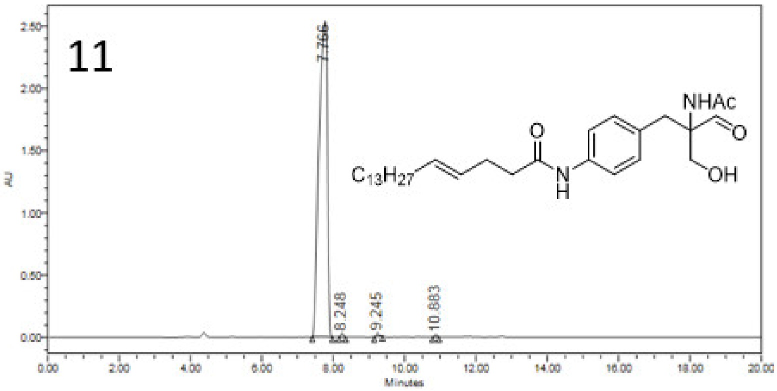

	RT	Area	% Area	Height
1	7.766	40820399	99.05	2537141
2	8.248	149268	0.36	28505
3	9.245	146188	0.35	28676
4	10.883	97386	0.24	17860

SAMPLE INFORMATION

Sample Name:	8129	Acquired By:	System
Sample Type:	Unknown	Sample Set Name	General method set_10
Vial:	9	Acq. Method Set:	General_A
Injection #:	1	Processing Method	General_A
Injection Volume:	20.00 ul	Channel Name:	W2489 ChA
Run Time:	20.0 Minutes	Proc. Chnl. Descr.:	W2489 ChA 254nm

Date Acquired: 12/9/2021 12:32:51 PM KST
Date Processed: 12/10/2021 1:31:41 PM KST

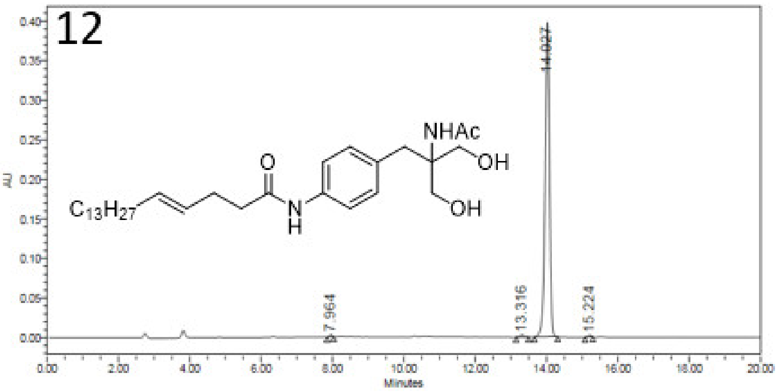

	RT	Area	% Area	Height
1	7.964	13300	0.39	2543
2	13.316	20589	0.61	2665
3	14.027	3352434	98.64	398629
4	15.224	12266	0.36	1895

SAMPLE INFORMATION

Sample Name:	8131	Acquired By:	System
Sample Type:	Unknown	Sample Set Name	General method set_10
Vial:	10	Acq. Method Set:	General_A
Injection #:	1	Processing Methoc	General_A
Injection Volume:	20.00 ul	Channel Name:	W2489 ChA
Run Time:	20.0 Minutes	Proc. Chnl. Descr.:	W2489 ChA 254nm
Date Acquired:	12/9/2021 1:23:21 PM KST		
Date Processed:	12/9/2021 2:20:23 PM KST		

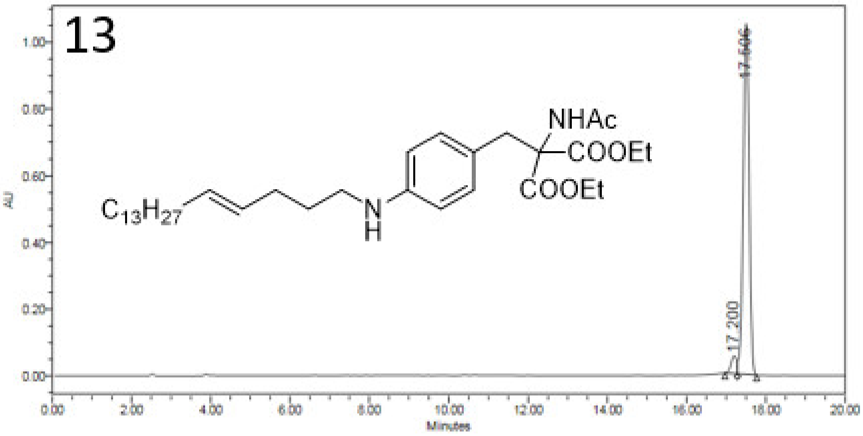

	RT	Area	% Area	Height
1	17.200	515290	4.51	53106
2	17.506	10905134	95.49	1053609

SAMPLE INFORMATION

Sample Name:	8132	Acquired By:	System
Sample Type:	Unknown	Sample Set Name	1
Vial:	39	Acq. Method Set:	General_A
Injection #:	1	Processing Method	General_A
Injection Volume:	10.00 ul	Channel Name:	W2489 ChB
Run Time:	20.0 Minutes	Proc. Chnl. Descr.:	W2489 ChB 280nm
Date Acquired:	12/10/2021 12:41:24 PM KST		
Date Processed:	12/10/2021 1:41:26 PM KST		

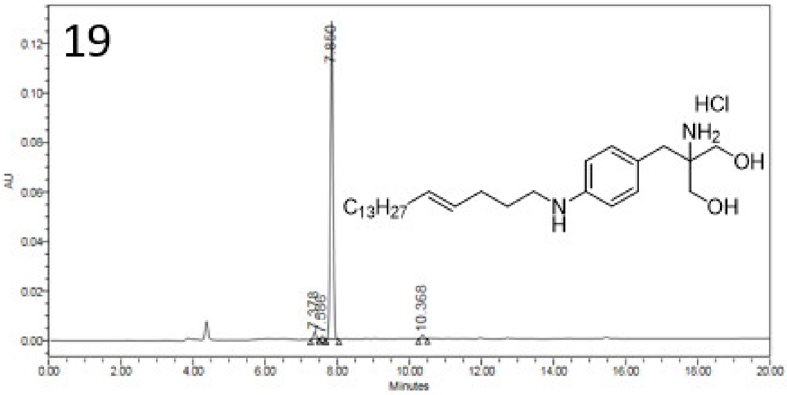

	RT	Area	% Area	Height
1	7.378	14322	1.96	2717
2	7.586	5011	0.69	1010
3	7.850	701466	96.16	127137
4	10.368	8660	1.19	1511

SAMPLE INFORMATION			
Sample Name:	8130	Acquired By:	System
Sample Type:	Unknown	Sample Set Name	General method set_10
Vial:	5	Acq. Method Set:	General_A
Injection #:	1	Processing Method	General_A
Injection Volume:	10.00 ul	Channel Name:	W2489 ChA
Run Time:	20.0 Minutes	Proc. Chnl. Descr.:	W2489 ChA 254nm
Date Acquired:	12/9/2021 6:04:06 PM KST		
Date Processed:	12/9/2021 6:27:33 PM KST		

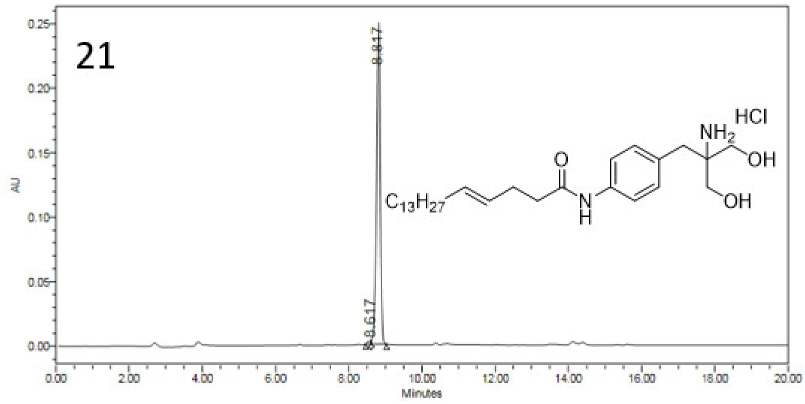

	RT	Area	% Area	Height
1	8.617	15540	0.88	2658
2	8.817	1745550	99.12	248011

HRMS

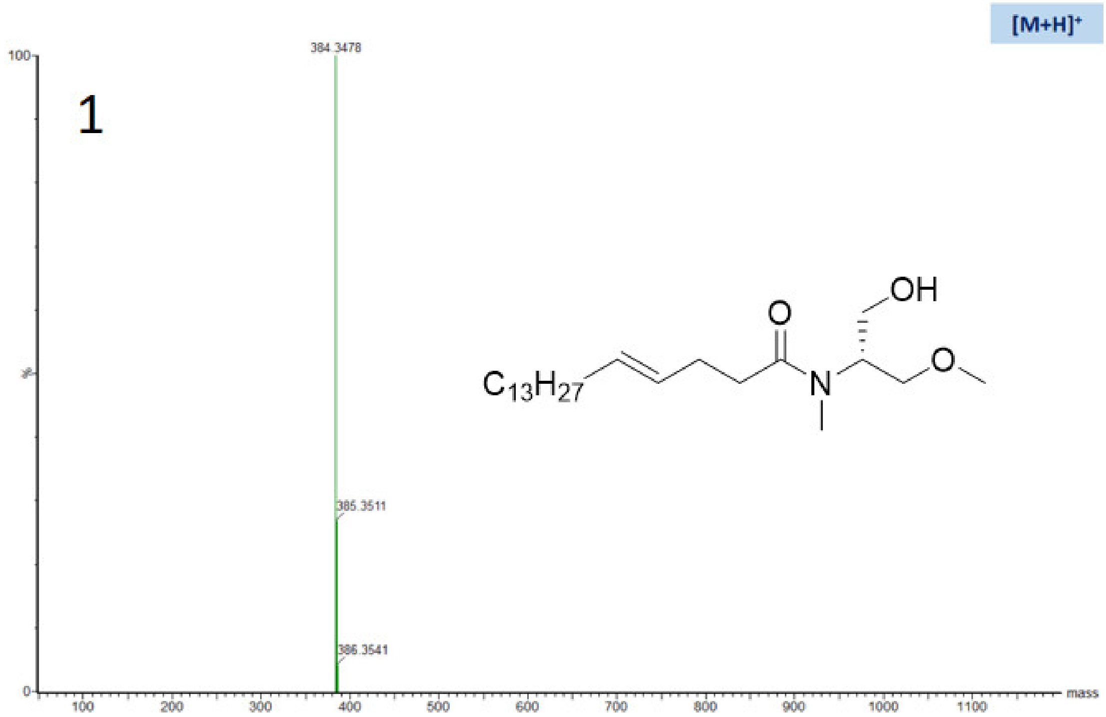
[M+H]+
100
1
384.3478
385.3511
386.3541
%
0
100 200 300 400 500 600 700 800 900 1000 1100
mass
C13H27
O
N
OH
O

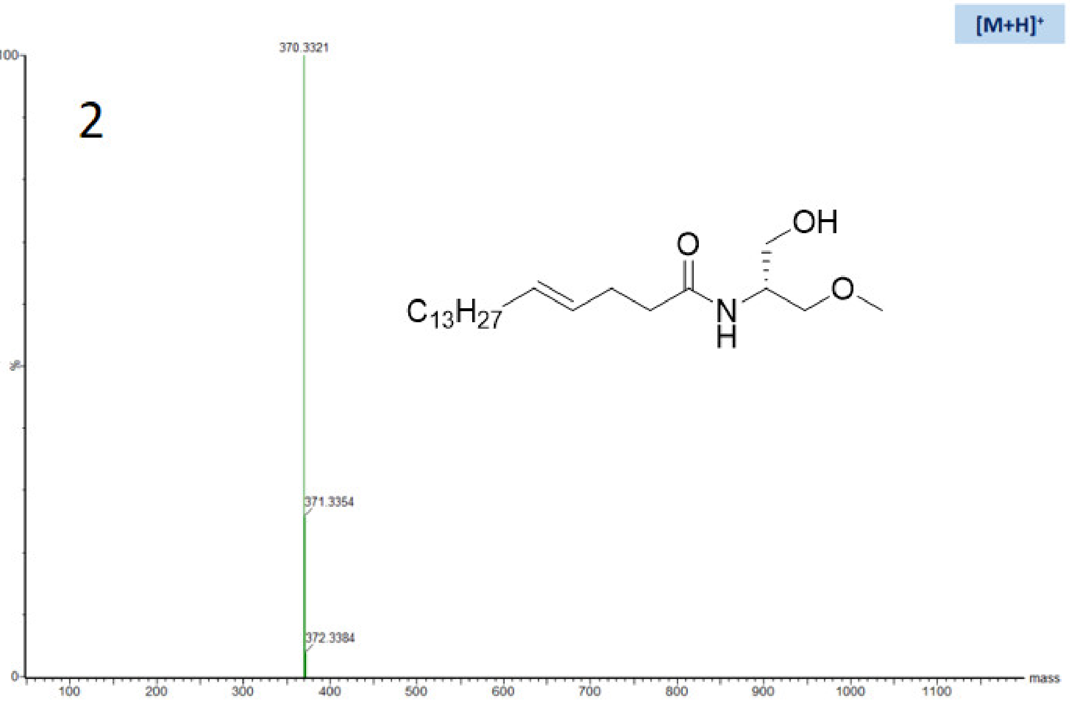
[M+H]+
100
2
370.3321
371.3354
372.3384
%
0
100 200 300 400 500 600 700 800 900 1000 1100
mass
C13H27
O
N
H
OH
O

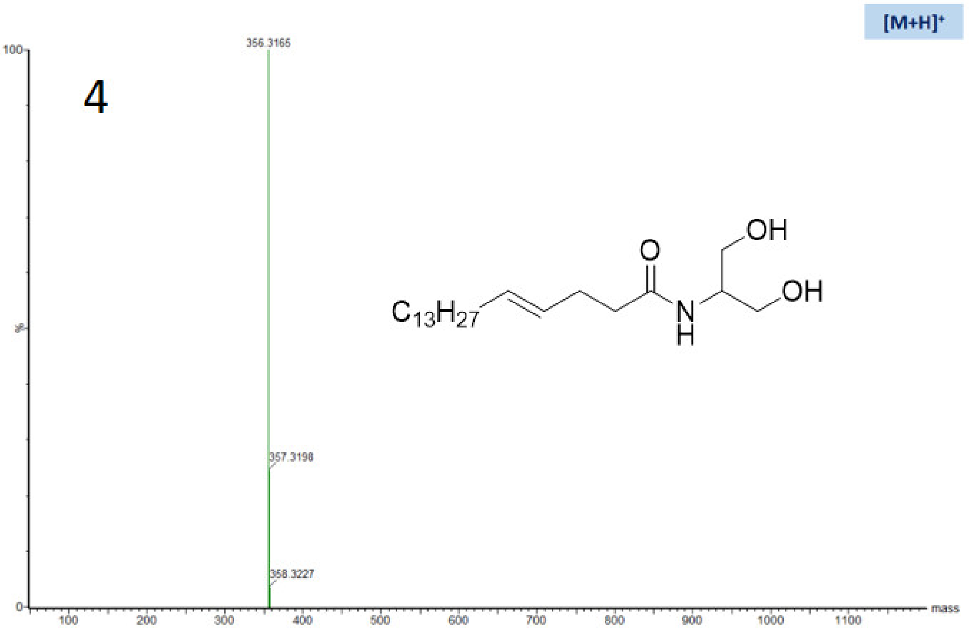

[M+H]+
4
356.3165
357.3198
358.3227
OH
O
OH
N
H
$C_{13}H_{27}$
mass

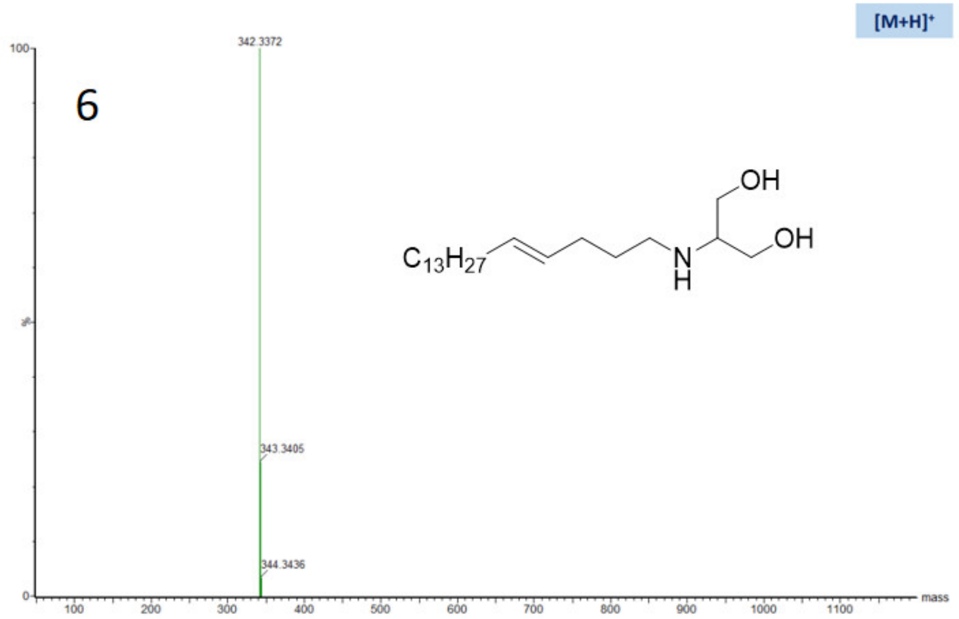

[M+H]+
6
342.3372
343.3405
344.3436
OH
OH
N
H
$C_{13}H_{27}$
mass

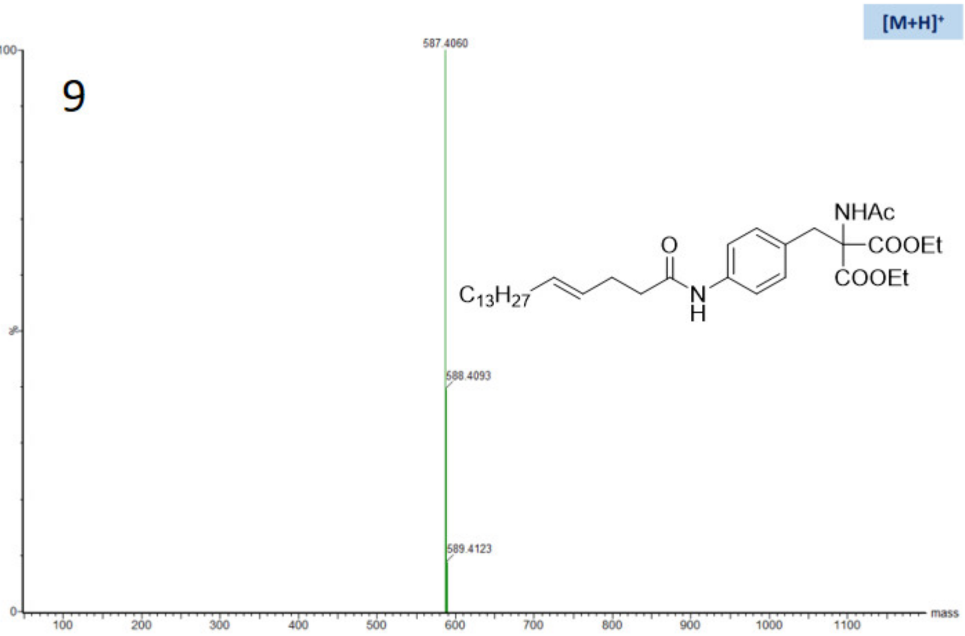
[M+H]+
9
587.4060
588.4093
589.4123
NHAc
COOEt
COOEt
O
N
H
C13H27
mass

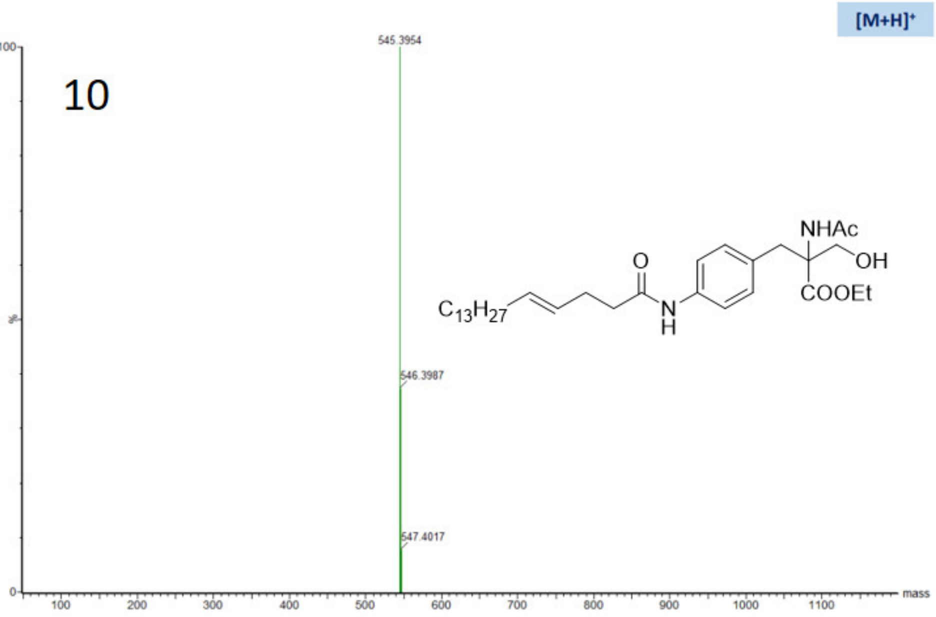
[M+H]+
10
545.3954
546.3987
547.4017
NHAc
OH
COOEt
O
N
H
C13H27
mass

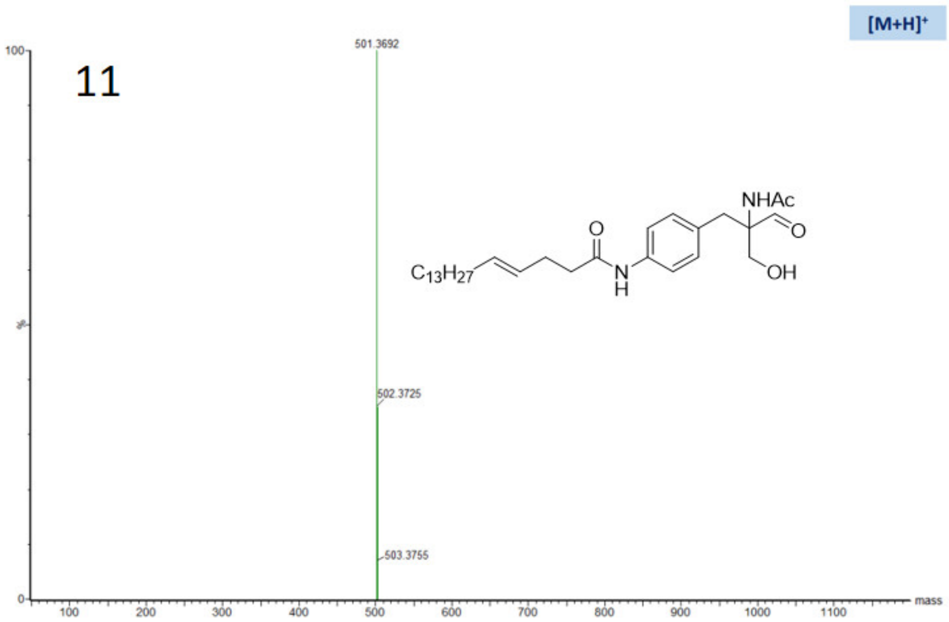
[M+H]+
11
501.3692
502.3725
503.3755
NHAc
O
O
N
H
OH
$C_{13}H_{27}$
mass

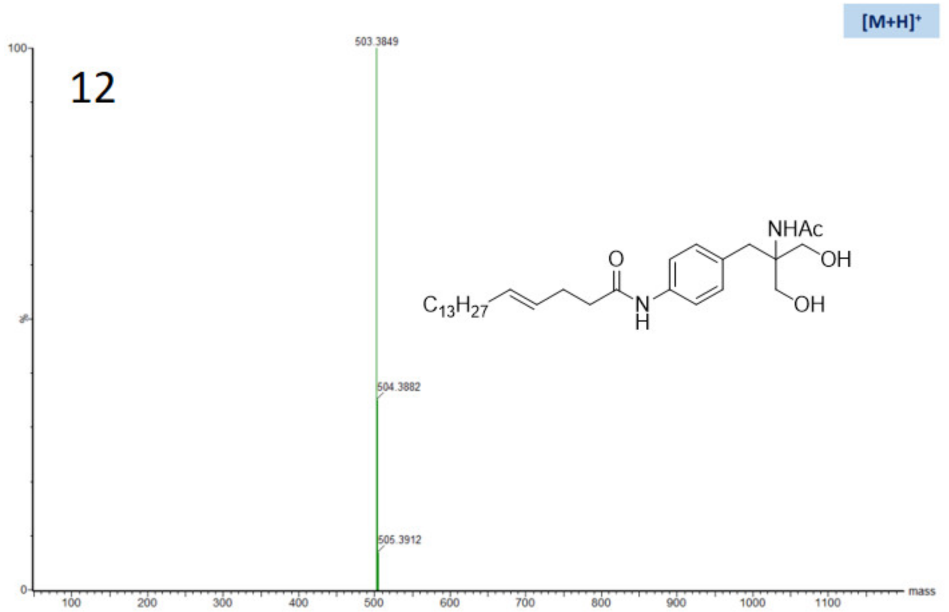
[M+H]+
12
503.3849
504.3882
505.3912
NHAc
O
OH
OH
N
H
$C_{13}H_{27}$
mass

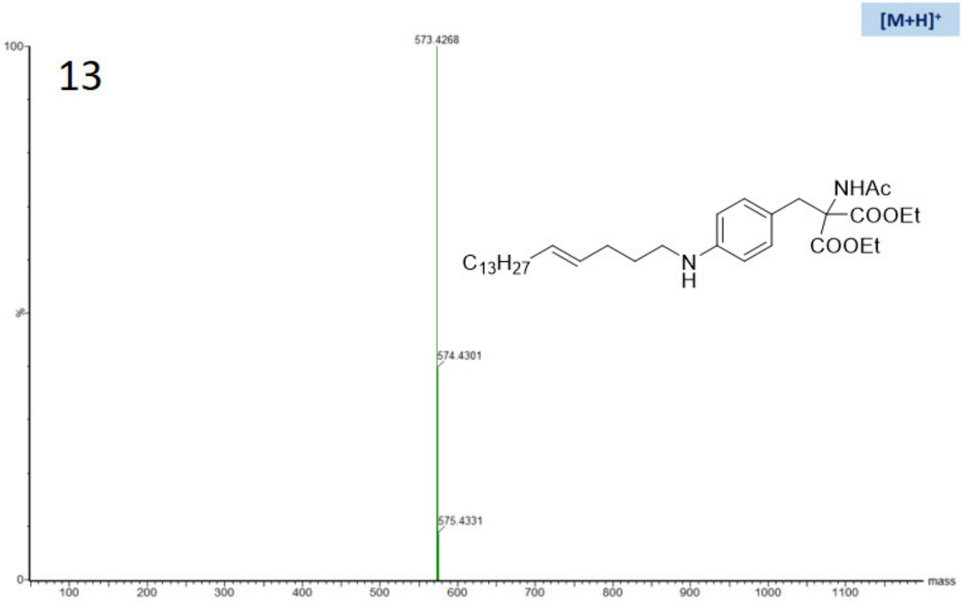

Isotope Modeling: KDS8132 (C28H50N2O2, Exact MS 446.3872)

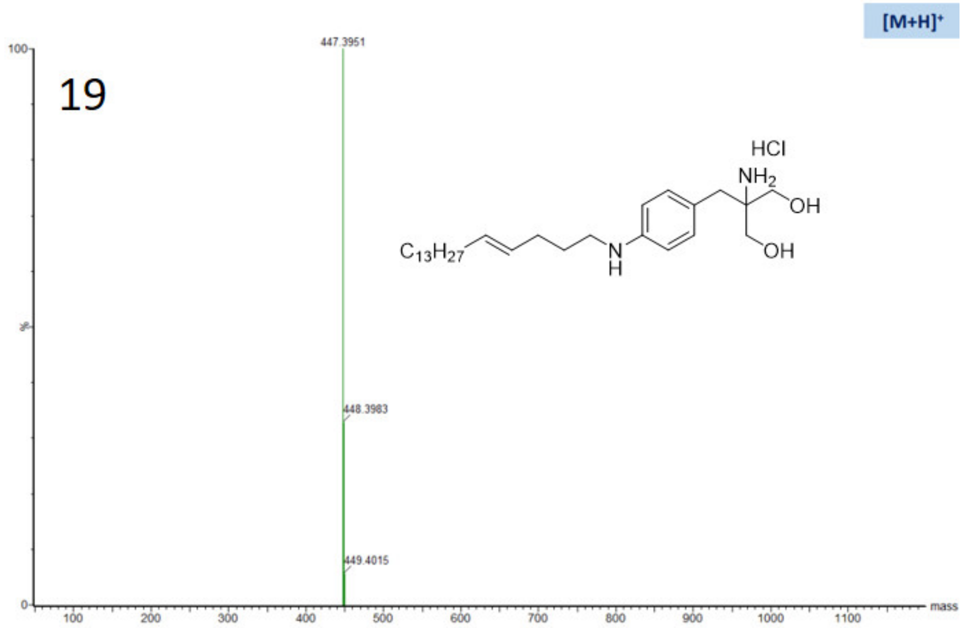

21

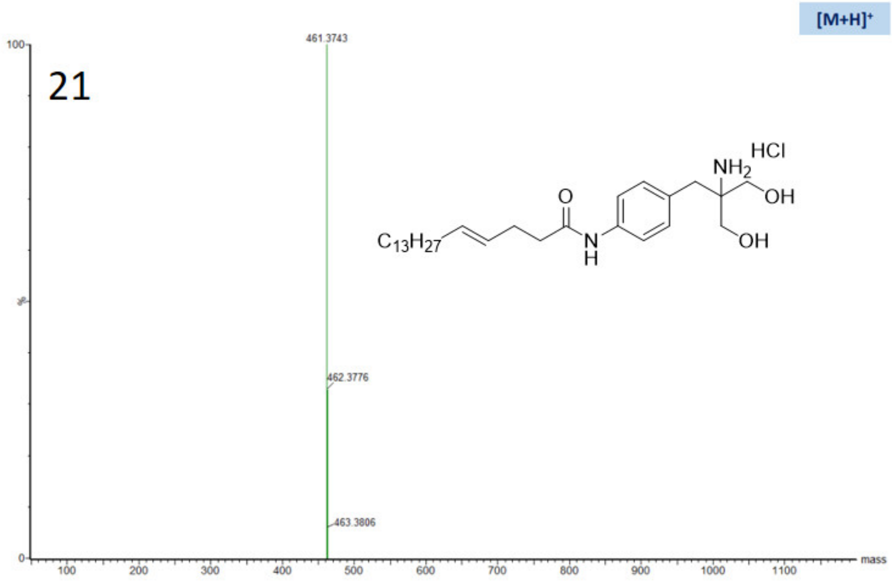

References

1. Xia, L.; Han, M-J.; Zhou, L.; Huang, A.; Yang, Z.; Wang, T.; Li, F.; Yu, L.; Tian, C.; Zang, Z.; Yang, Q-X.; Liu, C.; Hong, W.; Lu, Y.; Alfonta, L.; Wang, J. S-Click Reaction for Isotropic Orientation of Oxidases on Electrodes to Promote Electron Transfer at Low Potentials. *Angew*. *Chem*., *Int*. *Ed*. **2019**, 58, 16480–16484.
2. Gao, Y. R.; Guo, S. H.; Zhang, Z. X.; Mao, S.; Zhang, Y. L.; Wang, Y. Q.; Concise synthesis of (+)-serinolamide A. *Tetrahedron Lett*, **2013**, 54, 6511–6513.
